# MERS-CoV nsp1 regulates autophagic flux via mTOR signalling and dysfunctional lysosomes

**DOI:** 10.1080/22221751.2022.2128434

**Published:** 2022-10-26

**Authors:** Yujie Feng, Zhaoyi Pan, Zhihui Wang, Zhengyang Lei, Songge Yang, Huajun Zhao, Xueyao Wang, Yating Yu, Qiuju Han, Jian Zhang

**Affiliations:** Institute of Immunopharmaceutical Sciences, School of Pharmaceutical Sciences, Shandong University, Jinan, People’s Republic of China

**Keywords:** MERS-CoV, nsp1, autophagic flux, mTOR, lysosomes

## Abstract

Autophagy, a cellular surveillance mechanism, plays an important role in combating invading pathogens. However, viruses have evolved various strategies to disrupt autophagy and even hijack it for replication and release. Here, we demonstrated that Middle East respiratory syndrome coronavirus (MERS-CoV) non-structural protein 1(nsp1) induces autophagy but inhibits autophagic activity. MERS-CoV nsp1 expression increased ROS and reduced ATP levels in cells, which activated AMPK and inhibited the mTOR signalling pathway, resulting in autophagy induction. Meanwhile, as an endonuclease, MERS-CoV nsp1 downregulated the mRNA of lysosome-related genes that were enriched in nsp1-located granules, which diminished lysosomal biogenesis and acidification, and inhibited autophagic flux. Importantly, MERS-CoV nsp1-induced autophagy can lead to cell death *in vitro* and *in vivo*. These findings clarify the mechanism by which MERS-CoV nsp1-mediated autophagy regulation, providing new insights for the prevention and treatment of the coronavirus.

## Introduction

MERS-CoV is a highly pathogenic coronavirus that was first discovered in Saudi Arabia in 2012. MERS-CoV was seemingly transferred from infected dromedaries to humans, with a mortality rate of 35.6% [1]. Severe acute respiratory syndrome coronavirus (SARS-CoV), MERS-CoV, and SARS-CoV-2, which broke out in 2019 and caused a global pandemic, are all beta coronaviruses (β-CoVs) [2]. In addition to causing fever, headache, and fatigue, they can cause lung and kidney failure [2,3], seriously threatening human life [4]. Therefore, elucidating how coronavirus-encoded proteins interact with host factors is crucial in understanding the high infectivity and pathogenicity of these viruses, benefiting the development of strategies to prevent and treat coronaviruses.

MERS-CoV is enveloped RNA viruses that contain a single 5'-capped and 3'-polyadenylated RNA genome (∼30 kb) that codes for two large overlapping open reading frames (ORF1a and ORF1b) as well as a variety of structural and accessory proteins at the 3’ end [5]. Upon entry into host cells, ORF1a and ORF1b are translated and then processed by virus-encoded proteinases to produce functional nonstructural proteins (nsps) that take important part in viral infection and RNA genome replication [6]. It has reported that the M, NS4a, NS4b and nsp3 of MERS-CoV can antagonize interferon responses [7,8]. In addition, nsp12 and nsp13 of MERS-CoV play important roles in viral replication [9]. MERS-CoV nsp1 is the first viral nonstructural protein encoded by ORF1a. As a potent pathogenicity factor, we found previously nsp1 located in a novel ribonucleosome complex formed via liquid–liquid phase separation (LLPS), resulting in template-dependent cleavage and degradation of mRNAs in infected cells, thereby impairing the cellular metabolic processes [10].

Autophagy is a highly conserved degradation pathway in eukaryotes that maintains cell survival, material recycling, and homeostasis [11]. Normally, low autophagy levels are beneficial for maintaining a stable intracellular environment. When cells are subjected to various stress conditions, such as energy deprivation [12] or viral infection [13], autophagy can be activated to promote the initiation and nucleation of a crescent-shaped phagophore, which subsequently expands until it approaches the autophagosome [14]. Upon closure, autophagosomes fuse with vesicles originating from the endosomal compartment before forming degradative autolysosomes [15]. Several proteins that act at different steps of the autophagy pathway have been identified [16]. Many coronavirus-encoded proteins reportedly induce autophagy through different mechanisms. For highly pathogenic coronaviruses, nsp3, nsp4, and nsp6 of SARS-CoV can induce double-membrane vesicles [17], whereas ORF3a, ORF7a, M, and nsp6 of SARS-CoV-2 can promote LC3 punctate aggregation [18]. Furthermore, nsp3, nsp4, and nsp6 of MERS-CoV, as well as the accessory proteins ORF4b and ORF5a, can promote LC3-II accumulation [19,20]. The fusion of autophagosomes and lysosomes is mainly mediated by SNARE (soluble N-ethylmaleimide-sensitive factor attachment protein receptor) complexes, tether complexes, Rab molecules, and cytoskeleton components [21]. Viral infection can also affect autophagy by affecting lysosomal biogenesis and function. ORF8a of SARS-CoV promotes autophagic flux by promoting the nuclear translocation of transcription factor EB (TFEB) [22], whereas ORF7a of SARS-CoV-2 inhibits the degradation of autophagic contents by affecting the acidification of lysosomes [23].

Mammalian target of rapamycin (mTOR) is an evolutionarily conserved serine/threonine-protein kinase involved in cellular processes such as cell growth, cell cycle, cell survival, and autophagy [24]. AMP-activated protein kinase (AMPK) acts as a nutrient and energy sensor, sensing the level of intracellular ATP and maintaining the energy balance [25]. As ATP production mostly occurs in mitochondria, the site of oxidative phosphorylation (OXPHOS) [26], AMPK can be activated in the case of mitochondrial dysfunction, which in turn suppresses mTOR and maintains energy balance through various pathways, including autophagy, glycolysis, and mitochondrial homeostasis [27]. It has been reported that MERS-CoV infection can block autophagic flux [28]; our previous study demonstrated that MERS-CoV nsp1 disturbs mitochondrial function by selectively downregulating the mRNAs of OXPHOS-coding genes [10]. However, whether MERS-CoV nsp1 affects autophagy and its related mechanisms have not been elucidated.

This study revealed that MERS-CoV nsp1 induced autophagy by activating the AMPK-mTOR signalling pathway. Additionally, nsp1 affected lysosomal biogenesis and acidification, thereby inhibiting autophagic flux. Significantly, nsp1-induced autophagy led to cell death.

## Materials and methods

### Cell lines and cell culture

A549 and HEK 293 T cell lines were purchased from ATCC and grown in DMEM (HyClone, SH30022.01) supplemented with 10% fetal bovine serum (Biological Industries, 04-010-1A),100 U/mL penicillin, and 100 mg/mL streptomycin. Mouse embryonic fibroblasts (MEFs) were generated as previously described [29]. To induce nsp1 expression, 2 μg/mL doxycycline (DOX, Selleck, S5159) was used for 3 days. Cells were cultured in a humidified incubator with an atmosphere of 5% CO_2_ at 37 °C.

### Mice

According to previous methods [10], male and female mice (8–12 week) were exposed to drinking water supplemented with 2 mg/mL DOX for 21 days. All operations and experiments were performed according to the international guidelines concerning the care and treatment of experimental animals.

### Immunoblotting

Cells were lysed with RIPA lysis buffer (Beyotime, P0013D) containing 1% protease inhibitor. Following high-speed centrifugation (13,400 × g) for 15 min at 4 °C, the supernatant was collected, and its concentration was measured using the BCA method. Denatured proteins were separated by 8%, 10%, 12%, or 15% SDS-PAGE and then transferred to PVDF membranes. Membranes were blocked for 2 h and incubated with primary antibodies overnight at 4 °C, followed by incubation with HRP-conjugated secondary antibodies for 1 h. The signals were detected using a GelDoc XR+ imaging system (Bio-Rad). All protein signals were collected at different exposure times to ensure that the bands were not overexposed and were within the linear range for quantitative analysis.

### Immunofluorescence and confocal microscopy

For the detection of autophagosomes, a plasmid expressing EGFP-LC3 or mCherry-EGFP-LC3 was transfected in the presence or absence of nsp1 vectors. For other immunostaining assays, cells were fixed with 4% paraformaldehyde for 15 min at room temperature and blocked with Immunol Staining Blocking Buffer (Beyotime; P0102) and 5% goat serum (Beyotime, C0265) for 1 h at 37 °C. These cells were then stained with primary antibodies overnight at 4 °C and incubated with a fluorescent secondary antibody for 1 h after washing with PBS three times. DAPI (Beyotime, P0131) was used to stain the nucleus for 10 min. Images were captured using a confocal laser-scanning microscope. All image analyses were performed using Image J Software and Zeiss Auto-measure software.

### nsp1 granule sorting and mass spectrometry analysis

We proceeded with this according to previous studies [10]. Briefly, the cells were collected, washed with pre-cooled PBS, and then lysed in buffer (50 mM Tris, pH 7.4, 1 mM EDTA, 150 mM NaCl, 0.2% Triton X-100,100 U/mL ribonuclease inhibitor, protease inhibitor and phosphatase inhibitor). The nuclei were separated by centrifuging at 200 × g for 5 min. DNase I (4 U/mL; Beyotime, D7076) was used to eliminate any residual DNA contaminants. One part of the supernatant was added to agarose beads coated with the GFP antibody and incubated overnight at 4 °C, referred to as “Sorted.” The remaining supernatant was centrifuged at 10,000 × g for 10 min, defined as “Pre-sorted.” Proteins were eluted with sodium dodecyl sulfate buffer and analyzed by immunoblotting. For the mass spectrometry assay, protein strips were cut after SDS-PAGE gel separation and Coomassie brilliant blue staining.

### RNA sequencing

The mRNA-seq libraries were prepared following next-generation sequencing (NGS) protocols (Shanghai Personal Biotechnology) using the Illumina NovaSeq system.

### RNA immunoprecipitation-qPCR (RIP-qPCR) analysis

This procedure was performed according to a previous report [10]. Briefly, cells were lysed in lysis buffer (25 mM Tris-HCl, pH 7.5, 150 mM KCl, 2 mM EDTA, 0.5% NP-40, 1 mM NaF, 1 mM DTT, 100 U/mL ribonuclease inhibitor, protease inhibitor and protease inhibitor) for 10 min at 4 °C. The lysates were centrifuged, and the supernatant was incubated with nsp1 antibody-conjugated magnetic beads for 4 h at 4 °C and washed with buffer (50 mM Tris, 200 mM NaCl, 2 mM EDTA, 0.05% NP-40, 0.5 mM DTT, RNase inhibitor). To eliminate the remaining DNA and protein, DNase I was added and incubated for 10 min at 37 °C, and proteinase K (Invitrogen, 4333793) was added and incubated for 10 min at 55 °C. RNA was extracted using the TRIzol reagent and detected using RT-qPCR.

### Fluorescence in situ hybridization assay

Fluorescence *in situ* hybridization (FISH) assays were detected according to the protocol (RiboBio C10910). In brief, after different treatments, HEK 293 T cells were fixed and permeabilized in PBS containing 0.5% Triton X-100. Hybridization was performed overnight in a humidified chamber at 37 °C in the dark. Cy3 channels were used to detect signals, and all images were obtained using a confocal laser-scanning microscope.

### Histology and immunohistochemistry

Paraffin-embedded lung, kidney, and liver tissues were fixed with 4% paraformaldehyde, and the effect of nsp1 on tissue damage was detected by staining with hematoxylin and eosin (HE). The expression of p62 in lung and kidney tissues was detected by immunohistochemistry. Briefly, sections were prepared and blocked with 5% goat serum, followed by incubation with primary antibodies against p62 at 4 °C overnight. The sections were then washed and incubated with the mouse/rabbit streptomycin-biotin assay system (SP-9000; ZSGB-BIO, Beijing, China). The expression of p62 was visualized using 3,3′-diaminobenzidine tetrahydrochloride (ZLI-9017; ZSGB-BIO). Images were captured using an IX73 microscope (Olympus). At least three random fields were examined for each sample.

### Statistical analysis

GraphPad Prism version 8.0 was used for statistical analysis. Differences were analyzed using the Student’s t-test or two-way ANOVA for variables.

## Results

### MERS-CoV nsp1 triggers the accumulation of autophagosomes

**Figure 1.**
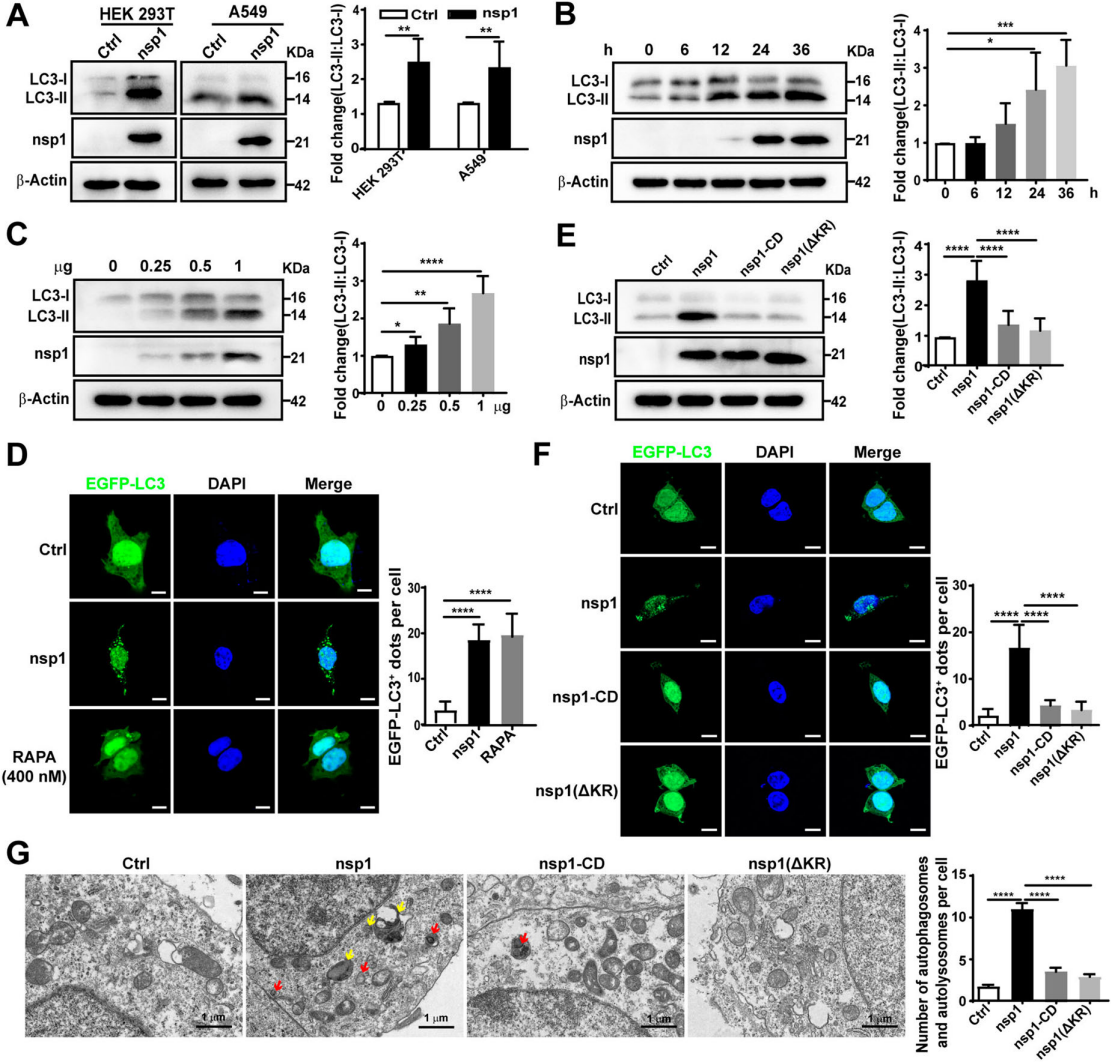
nsp1 promotes the accumulation of autophagosomes. (A) HEK 293 T and A549 cells were transfected with the indicated plasmids for 36 h, after which cell lysates were analyzed by immunoblotting. HEK 293T were transfected with the nsp1 plasmid for the indicated times (B) or different doses (C), and cell lysates were analyzed by immunoblotting. (D) HEK 293 T cells were co-transfected with the indicated plasmids for 36 h, after which confocal microscopy was performed to analyze the EGFP-LC3^+^ dots; RAPA was used to induce autophagy. (E) HEK 293 T cells were transfected with the indicated plasmids for 36 h, and cell lysates were analyzed by immunoblotting. (F) HEK 293T cells were co-transfected with the indicated plasmids for 36 h, and EGFP-LC3^+^ dots were analyzed by confocal microscopy. (G) HEK 293 T cells were transfected with the indicated plasmids for 36 h, the accumulation of autophagosomes and autolysosomes was analyzed via TEM. Red, autophagosomes; yellow, autolysosomes. Quantitative analysis of LC3-II:LC3-I ratios was performed with Image J software. The average value in Ctrl-transfected cells was normalized to 1 in the immunoblotting quantitative calculations. The number of EGFP-LC3^+^ dots in each cell was counted with Image J software, and at least twenty cells were included for each group. The scale bars are all 10 μm. Data are presented as the mean ± SEM from at least three independent experiments (*p < 0.05, **p < 0.01, ***p < 0.001, and ****p < 0.0001).

To determine whether MERS-CoV nsp1 (referred to as nsp1 unless the viral origin is stated) regulates autophagy, we first explored the effect of nsp1 on autophagosome formation. As the LC3-II:LC3-I ratio is regarded as an accurate proxy of autophagic activity [13], we assessed the conversion of endogenous LC3-I to LC3-II via immunoblotting. Compared to the control (Ctrl), nsp1 significantly increased the LC3-II:LC3-I ratio (Figure 1A) in a time- (Figure 1B and S1A) and dose-dependent manner (Figure 1C), indicating a continuous increase in autophagosome formation. Furthermore, nsp1 promoted the formation of EGFP-LC3-labelled dots compared with Ctrl, which was similar to rapamycin (RAPA) (Figure 1D and S1B).

As an endonuclease, nsp1 can selectively downregulate the mRNAs of host cells, depending on 146R and 147 K [30]. To determine whether the effect of nsp1 on autophagosome accumulation depends on its endonuclease activity, we constructed nsp1 with R146A and K147A mutations (nsp1-CD) and nsp1 with R146 and K147 deletions (nsp1-ΔKR). Compared with nsp1, nsp1-CD and nsp1-ΔKR did not significantly influence the LC3-II:LC3-I ratio (Figure 1E) or the number of EGFP-LC3-labelled dots (Figure 1F). To directly visualize autophagosome formation in nsp1-overexpressing cells, we used transmission electron microscopy (TEM) to observe the cellular ultrastructure. Autophagosomes with double membrane vacuoles contain cytoplasmic contents, and eventually fused with lysosomes to form autolysosomes. Late autolysosomes typically have only one limiting membrane containing cytoplasmic material and/or organelles at various stages of degradation [31]. Statistical analysis demonstrated that nsp1 increased the accumulation of autophagosomes (red arrows) and autolysosomes (yellow arrows) compared to Ctrl, whereas nsp1-CD and nsp1-ΔKR did not show a significant influence (Figure 1G). These data demonstrate that nsp1 can induce autophagosome accumulation depending on its endonuclease activity.

### nsp1 induces autophagy but inhibits autophagic flux

**Figure 2.**
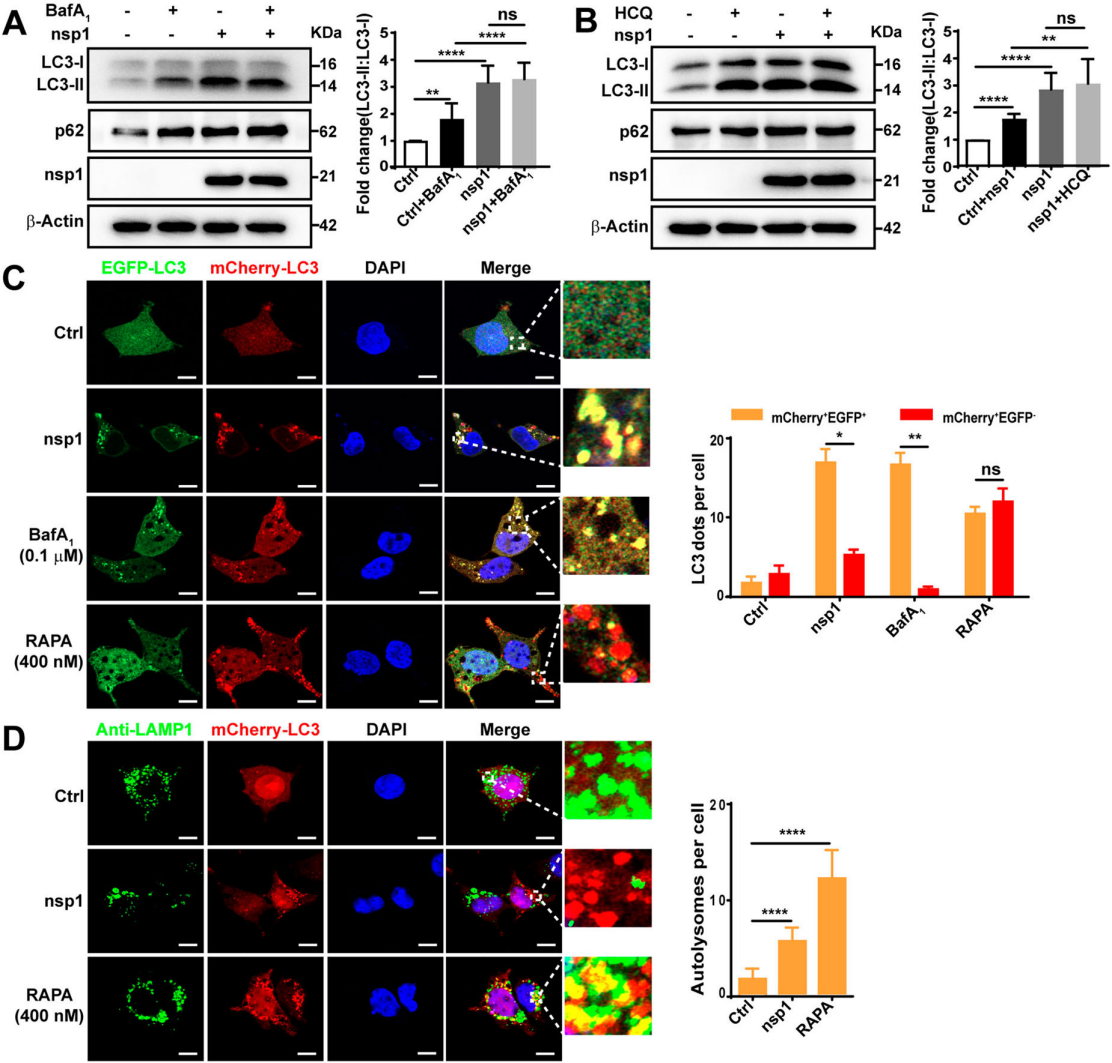
nsp1 induces autophagy but inhibits autophagic flux. HEK 293 T cells were transfected with the indicated plasmids for 30 h, then treated with BafA_1_ (A) or HCQ (B) for 6 h. Cell lysates were analyzed by immunoblotting. (C) HEK 293T cells were co-transfected with mCherry-EGFP-LC3 and the indicated plasmids for 36 h. Meanwhile, HEK 293T cells transfected with mCherry-EGFP-LC3 for 30 h and then treated with RAPA or BafA_1_ for 6 h, which were used as the controls for autophagy induction and the inhibition of autolysosome maturation, respectively. Autophagosomes (mCherry^+^EGFP^+^) and autolysosomes (mCherry^+^EGFP^-^) were analyzed by confocal microscopy. (D) HEK 293 T cells were co-transfected with mCherry-LC3 and indicated plasmids for 36 h, the co-localization of LAMP1 and mCherry-LC3 was analyzed by confocal microscopy. Quantitative analyses of LC3-II:LC3-I and p62: β-Actin were performed with Image J software. The average value in Ctrl cells was normalized to 1 in the immunoblotting quantitative calculations. The number of autophagosomes and autolysosomes in each cell was counted with Image J software, and at least twenty cells were included in each group. The scale bars are all 10 μm. Data are presented as the mean ± SEM from at least three independent experiments (*p < 0.05, **p < 0.01, and ****p < 0.0001).

The accumulation of autophagosomes is an intermediate process within the autophagic flux, reflecting the balance between the rates of generation and conversion into autolysosomes. Thus, nsp1-induced the accumulation of autophagosomes may be due to increased formation or decreased degradation. To clarify this, we used two autophagy inhibitors, bafilomycin A1 (BafA_1_) and hydroxychloroquine (HCQ), which can suppress autophagic flux. Compared with Ctrl, the LC3-II:LC3-I ratio was increased upon HCQ/BafA_1_ treatment (Figure 2A and B; lanes 1 and 2) and nsp1-expression (Figure 2A and B; lanes 1 and 3). Higher LC3-II:LC3-I ratios were detected in nsp1-expressing cells than Ctrl upon HCQ/BafA_1_ treatment (Figure 2A and B; lanes 2 and 4), indicating that nsp1 induced autophagy. In addition, the LC3-II:LC3-I ratio in nsp1-overexpressing cells were not significantly influenced by treatment with or without HCQ/BafA_1_ (Figure 2A and B; lanes 3 and 4), indicating that nsp1 saturated the function of HCQ/BafA_1_ in blocking autophagic flux.

SQSTM1/p62 is a ubiquitin-binding protein that binds LC3 for cargo recruitment and degradation [32]. However, no degradation of p62 in nsp1-overexpressing cells was observed in our study (Figures 2A and B), further suggesting that nsp1 disturbs the autophagic flux. Next, we used the autophagy flux reporter mCherry-EGFP-LC3 to monitor the dynamics of autophagic flux. When autophagy is active, the EGFP signal disappears in response to low lysosomal pH; therefore, the fusion of autophagosomes and acidic lysosomes causes autolysosomes to exhibit red fluorescence. The redder the fluorescence, the smoother the autophagic flux. Compared with Ctrl, most of the LC3-positive autophagic vacuoles were red in RAPA-treated cells; however, similar to BafA_1_-treated cells, many yellow autophagic vacuoles were observed in nsp1-overexpressing cells (Figure 2C), indicating that nsp1 blocked the autophagic flux. Finally, lysosomes were tracked by lysosome-associated membrane protein 1 (LAMP1), and the co-localization of LAMP1 and LC3 was analyzed. We found that the co-localization of LAMP1 and LC3 was significantly reduced in nsp1-overexpressing cells relative to RAPA-treated cells (Figure 2D and S1C). These results demonstrate that nsp1 induces autophagy but inhibits autophagic activity.

### nsp1 affects mTOR signalling and promotes autophagy induction

**Figure 3.**
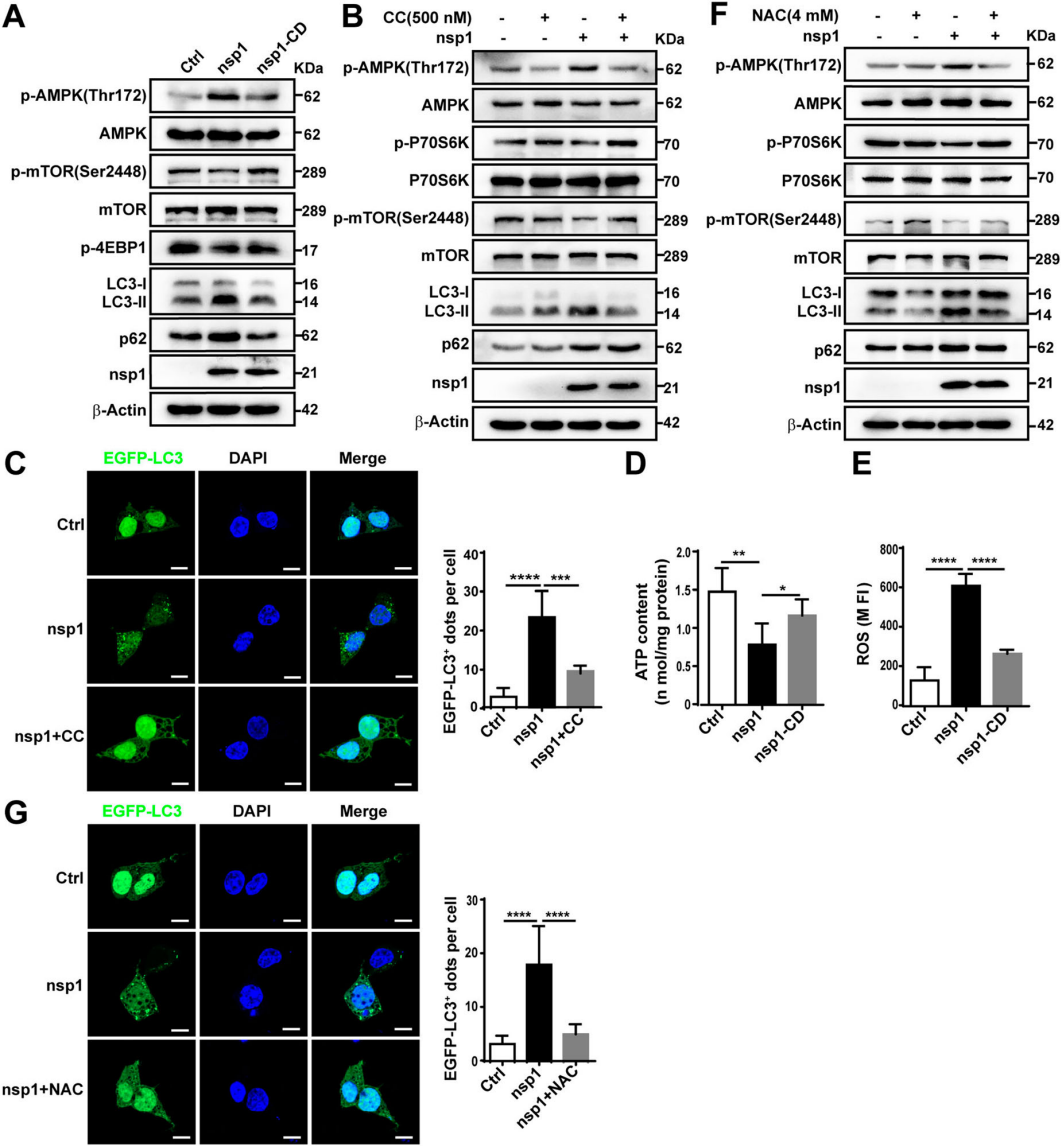
nsp1 induces autophagy through the AMPK-mTOR signalling pathway. (A) HEK 293 T cells were transfected with the indicated plasmids for 36 h, and cell lysates were analyzed by immunoblotting. (B and C) HEK 293 T cells were pretreated with CC for 2 h and then transfected with the indicated plasmids for another 36 h, cell lysates were evaluated by immunoblotting (B), and EGFP-LC3^+^ dots were observed by confocal microscopy (C). (D) HEK 293 T cells were transfected with the indicated plasmids for 36 h, after which the ATP levels were measured using a luminescence assay system. (E) HEK 293 T cells were transfected with the indicated plasmids for 36 h, and the intracellular ROS levels were examined by DCFH-DA assay. (F) and (G) HEK 293 T cells were transfected with the indicated plasmids for 36 h in the absence or presence of NAC; cell lysates were analyzed by immunoblotting (F), and EGFP-LC3^+^ dots were visualized by confocal microscopy (G). The number of EGFP-LC3^+^ dots in each cell was counted with Image J software, and at least twenty cells were included for each group. The scale bars are all 10 μm. Data are presented as the mean ± SEM from at least three independent experiments (*p < 0.05, **p < 0.01, ***p < 0.001, and ****p < 0.0001).

To clarify whether nsp1 induces autophagy via the canonical autophagy pathway, the important regulatory molecules Beclin1 and ATG5 were silenced. BECN1 and ATG5 knockdown resisted the nsp1-induced elevation of LC3B-II:LC3-I (Figure S2A and S2B), suggesting that nsp1 promoted autophagosome formation through the canonical autophagy pathway. mTOR is a key regulator of the canonical autophagy pathway. We found that nsp1 induced AMPK (T172) activation, accompanied by the inhibition of mTOR (S2448) signalling (Figure 3A), depending on the endonuclease activity of nsp1. This was distinct from RAPA treatment, which inhibited mTOR signalling by directly binding to mTOR without affecting AMPK (Figure S2C). However, nsp1 had no obvious influence on the phosphorylation of Akt and ERK1/2 (Figure S2D). Furthermore, pretreatment with compound C (CC), an AMPK inhibitor, significantly abrogated the nsp1-mediated accumulation of LC3-II (Figure 3B) and the increase in EGFP-LC3 dots (Figure 3C), implying that nsp1 induced autophagy by activating AMPK and inhibiting mTOR.

AMPK is pivotal to metabolism and mitochondrial homeostasis. In general, AMPK is activated in response to energy stress by sensing increases in the AMP:ATP and ADP:ATP ratios, subsequently restoring the energy balance through the inhibition of anabolism and the promotion of catabolism, processes that consume and generate ATP, respectively [25,26]. In addition, ROS can activate the AMPK signalling pathway to maintain the stability of oxidative metabolism [33]. Compared with Ctrl, nsp1-overexpression resulted in lower oxygen consumption rates (Figure S2E) and ATP production (Figure 3D), restored by nsp1-CD. Meanwhile, we found nsp1 increased intracellular ROS levels (Figure 3E) depending on its endonuclease activity. N-acetyl-L-cysteine (NAC), a ROS scavenger, partially suppressed the nsp1-induced LC3-II:LC3-I ratio (Figure 3F) and the number of EGFP-LC3 dots (Figure 3G). These findings reveal that nsp1 can disturb mitochondrial respiratory function, resulting in a decrease in ATP synthesis and an increase in ROS production, ultimately inducing autophagy by activating AMPK and inhibiting mTOR.

### nsp1 causes aberrant and dysfunctional lysosomes

**Figure 4.**
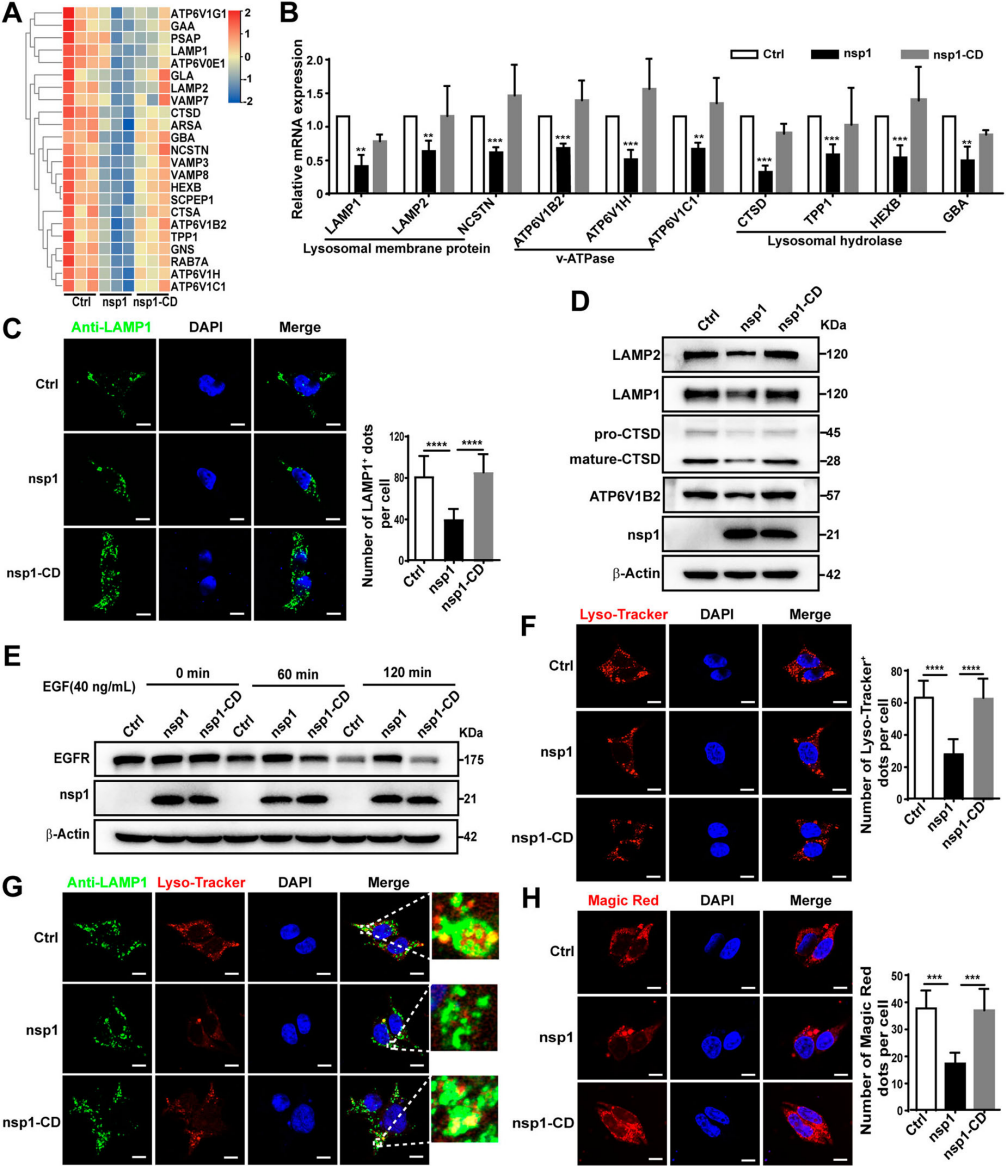
nsp1 impairs lysosomal biogenesis and acidification. (A) Heat map of downregulated lysosome-related genes by nsp1. (B) HEK 293 T cells were transfected with the indicated plasmids for 24 h, and the transcription levels of the indicated genes were analyzed by RT-qPCR. (C) HEK 293 T cells were transfected with the indicated plasmids for 36 h, then fixed and immunostained by an anti-LAMP1 antibody, and further analyzed by confocal microscopy. (D) HEK 293 T cells were transfected with the indicated plasmids for 36 h, and cell lysates were analyzed by immunoblotting. (E) HEK 293 T cells were transfected with the indicated plasmids for 36 h, then treated with EGF (40 ng/mL) for 0, 60, and 120 min; EGF-induced EGFR degradation was analyzed by immunoblotting. (F) HEK 293 T cells were transfected with the indicated plasmids for 36 h and then treated with Lyso-Tracker for 40 min and analyzed by confocal microscopy. (G) HEK 293 T cells were transfected with the indicated plasmids for 36 h, and the co-localization of LAMP1 and Lyso-Tracker was analyzed by confocal microscopy. (H) HEK 293 T cells were transfected with the indicated plasmids for 36 h, after which active cathepsin B was detected by Magic Red dye. The number of LAMP1^+^, Lyso-Tracker^+^, and Magic Red dots in each cell were counted with Image J software, and at least twenty cells were included for each group. The scale bars are all 10 μm. Data are presented as the mean ± SEM from at least three independent experiments (**p < 0.01, ***p < 0.001, and ****p < 0.001).

To further clarify the mechanism by which nsp1 inhibits autophagic flux, we performed a Co-IP/MS analysis. A key autophagosome-lysosome fusion protein, Myosin VI, was found in all the proteins that interacted with nsp1 (Figure S3A). Myosin VI can act as an adaptor that binds to the autophagy receptor, which interacts with Tom 1 on the endosomes or lysosomes, facilitating the fusion of autophagosomes and lysosomes [34]. However, the interaction between nsp1 and Myosin VI did not influence the interaction of Myosin VI with Tom1 by Co-IP analysis (Figure S3B), indicating that it is not the mechanism for the inhibition of autophagic flux induced by nsp1. Next, using RNA-Seq analysis (Figure 4A and Figure S4A; Table S2) and qPCR, we found that nsp1 decreased the mRNA levels of lysosome-related proteins, including membrane proteins (LAMP1, LAMP2, NCSTN), v-ATPase (ATP6V1B2, ATP6V1H, ATP6V1C1), acid enzyme-related proteins (CTSD, TPP1, HEXB, GBA) (Figure 4B). Meanwhile, nsp1 also decreased the mRNA levels of other lysosome-related proteins, like SNAREs, RAB7A, VPS41, which is associated with membrane fusion (Figure S4B). Therefore, we analyzed whether nsp1 influences lysosomal biogenesis. Confocal microscopy showed that the number of LAMP1-labelled lysosomes in nsp1-overexpressing cells was significantly reduced compared to Ctrl (Figure 4C), accompanied by the reduced expression of LAMP1, LAMP2, ATP6V1B2 and CTSD (Figure 4D), depending on the endonuclease activity of nsp1. Next, we explored whether nsp1 affected lysosomal degradation using an epidermal growth factor receptor (EGFR) degradation assay. As shown in Figure 4E, EGFR levels gradually decreased in Ctrl and nsp1-CD-treated cells along with EGF treatment, whereas nsp1-overexpressing cells exhibited resistance to EGFR degradation, indicating that nsp1 inhibited lysosomal degradation. Lysosomes must maintain an acidic pH for the degradative activity of hydrolases, such as cathepsins. Using Lyso-Tracker, a dye for labelling acidic vesicles, we found that nsp1 strongly inhibited Lyso-Tracker staining, where the number of Lyso-Tracker-positive dots was significantly reduced (Figure 4F). Significantly, most LAMP1-positive dots showed weak colocalization with Lyso-Tracker (Figure 4G), and the activity of lysosomal hydrolase cathepsin B was severely reduced in nsp1-overexpressing cells (Figure 4H). These results indicated that nsp1 inhibits autophagic flux by impairing lysosomal biogenesis and acidification.

### nsp1 influences lysosome-related genes through its endonuclease activity

**Figure 5.**
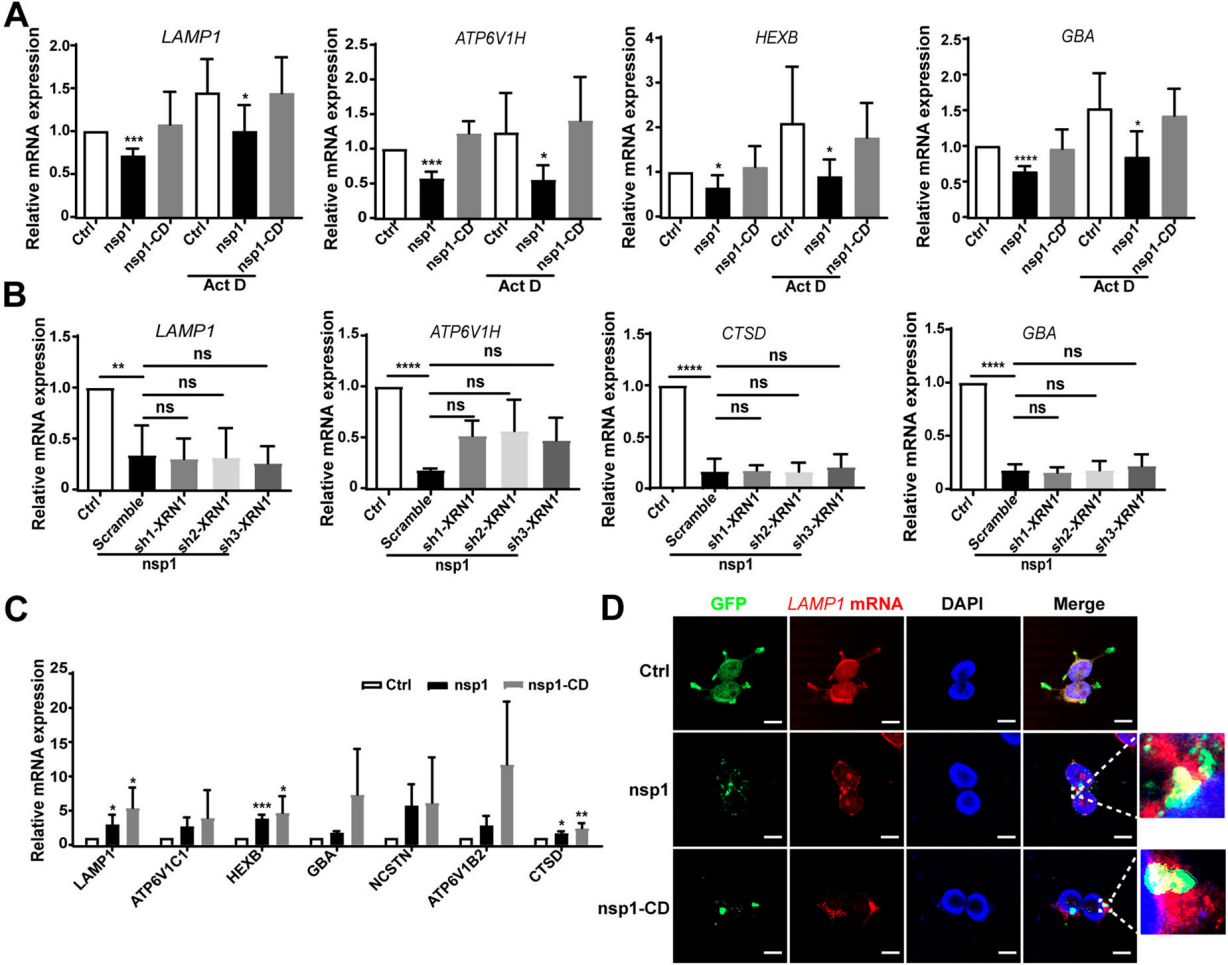
nsp1 inhibits lysosomal biogenesis and acidification through its endonuclease activity. (A) HEK 293 T cells were transfected with the indicated plasmids for 12 h, and then these cells were incubated in the absence or presence of Act D (2 μg/mL) for 24 h; mRNA levels of the indicated genes were analyzed by RT-qPCR. (B) Indicated plasmids were transfected into Scramble- or sh-XRN1-treated HEK293T cells; after 36 h, the mRNA levels of the indicated genes were analyzed by RT-qPCR. (C) HEK293T cells were transfected with the indicated plasmids for 36 h; the mRNAs that nsp1 directly bound to were detected by RIP-qPCR. (D) HEK 293 T cells were transfected with the indicated plasmids for 36 h, and the co-localization of *LAMP1* mRNA and nsp1 was analyzed by RNA-FISH. Scale bar: 10 μm. Data are presented as the mean ± SEM from at least three independent experiments (*p < 0.05, **p < 0.01, ***p < 0.001, and ****p < 0.001).

nsp1 may decrease the mRNA levels of lysosome-related genes by suppressing the transcription of upstream transcription factors and/or promoting mRNA degradation. TFEB, a member of the MiTF/TFE transcription factor family, is a major regulator of autophagy and lysosomal biogenesis [35]. We found that nsp1 did not significantly affect the mRNA (Figure S5A) or protein levels (Figure S5B) of TFEB compared to Ctrl. In contrast, nsp1 significantly increased the nuclear levels and localization of TFEB (Figure S5C and S5D). Generally, the nuclear translocation of TFEB promotes lysosome-related gene expression; therefore, these results could not explain the decrease in lysosomal proteins caused by nsp1. In addition, upon treatment with actinomycin D (Act D), which interferes with the transcriptional process, we found that nsp1 still reduced the mRNA levels of lysosome-related protein genes compared to the Ctrl and nsp1-CD cells (Figure 5A and S6A); this suggests that nsp1 induced the downregulation of lysosome-related gene mRNAs independent of the transcription of upstream transcription factors.

mRNAs can be degraded by host ribonucleases and/or nsp1, which act as endonucleases. XRN1, a highly efficient host exonuclease, can cleave 5’ monophosphates from the 5’ to 3’ direction, mediating mRNA cleavage in many viral infections [36]. However, we found that nsp1 still decreased the expression of lysosome-related genes in *XRN1*-knockdown cells compared to Ctrl (Figure 5B). Our previous study suggested that nsp1 was distributed as granules in cells and specifically cleaved the mRNAs of OXPHOS protein genes [10]. To investigate whether nsp1 downregulates the mRNA of lysosome-related genes in the same manner, we used a GFP antibody to isolate nsp1-located granules for MS detection. We found a large proportion of RNA-binding proteins in the nsp1-located granules (Figure S6B and Table S3). Furthermore, RIP-qPCR revealed that nsp1 was directly bound to the mRNA of lysosome-related genes such as *LAMP1, ATP6V1B2, HEXB*, etc. which was more evident in nsp1-CD cells (Figure 5C and Figure S6C). The co-localization of *LAMP1* mRNA and nsp1 was also observed using RNA-FISH (Figure 5D and S6D). These results indicate that nsp1 can bind to the mRNA of lysosome-related genes that are enriched in nsp1-located granules, and then cleave these mRNAs via endonuclease activity.

### nsp1-induced autophagy impairs cell viability

**Figure 6.**
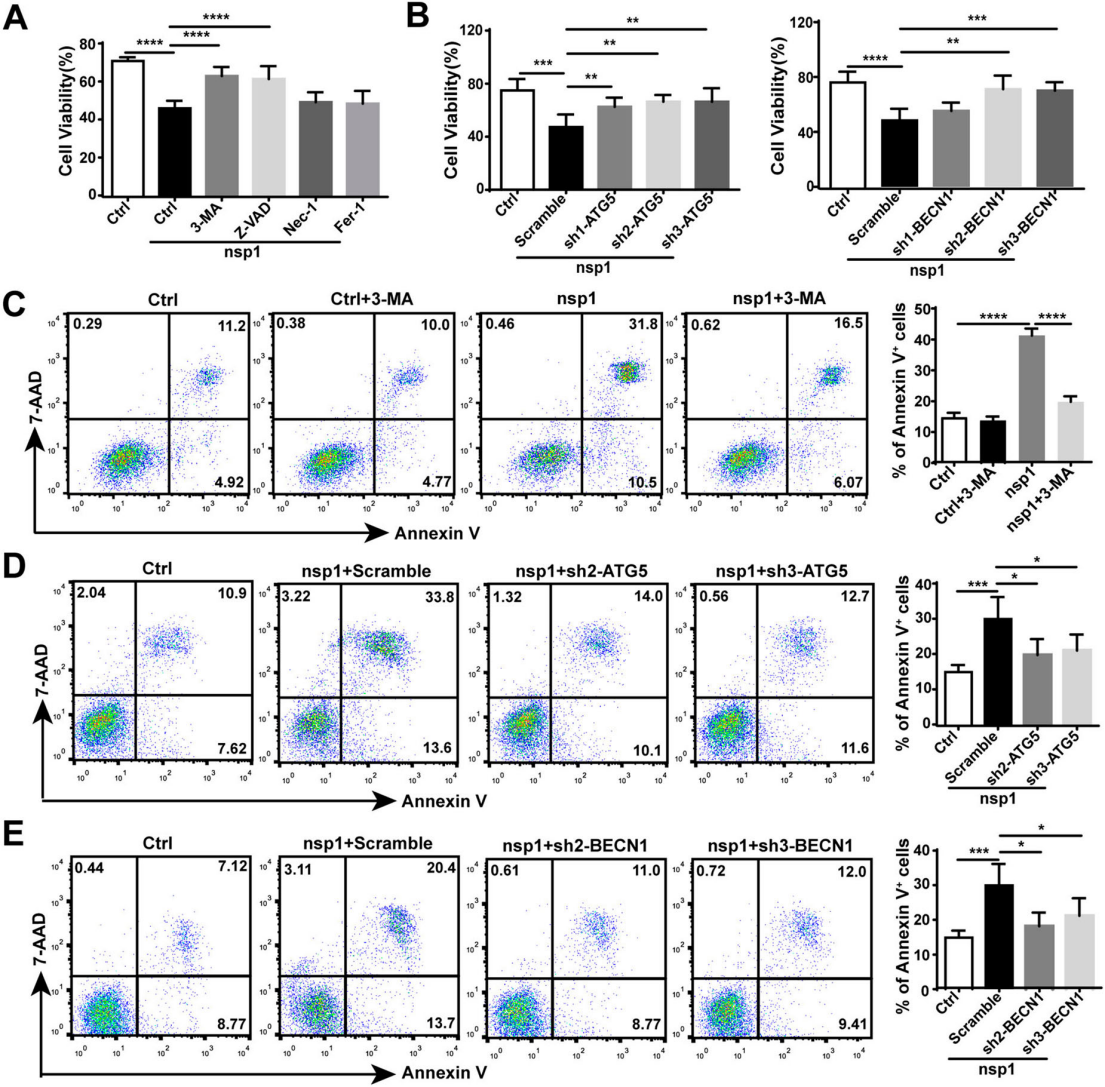
nsp1-induced autophagy impairs cell viability. (A) HEK 293 T cells were transfected with the indicated plasmids in the presence or absence of 3-MA, Z-VAD, Nec-1, and Fer-1 for 36 h; the cell viability was determined by CCK8 assay. (B) The indicated plasmids were transfected into Scramble- or ATG5/BECN1-knockdown HEK 293 T cells. After 36 h, the cell viability was determined by CCK8 assay. (C) HEK 293 T cells were transfected with the indicated plasmids in the presence or absence of 3-MA, and the apoptosis rate was determined by flow cytometry. The indicated plasmids were transfected into Scramble- or (D) ATG5/(E) BECN1-knockdown HEK293T cells for 36 h, and the apoptosis rate was determined by flow cytometry. Data are presented as the mean ± SEM from at least three independent experiments (*p < 0.05, **p < 0.01, ***p < 0.001, and ****p < 0.001).

To clarify the significance of nsp1-induced autophagy, we analyzed the influence of nsp1 on cell viability. Compared with Ctrl, nsp1 inhibited cell viability, which could be recovered by treatment with the autophagy inhibitor 3-MA or the apoptosis inhibitor Z-VAD, but not the necroptosis inhibitor Nec-1 or the ferroptosis inhibitor Fer-1 (Figure 6A), suggesting that autophagy and apoptosis might be the main forms of nsp1-induced cell death. Next, we used sh-ATG5/BECN1 to confirm the effect of nsp1-induced autophagy on cell viability. Results showed that sh-ATG5/BECN1 recovered the nsp1-induced impairment of cell viability compared with Scramble (Figure 6B). Simultaneously, 3-MA or sh-ATG5/BECN1 treatment significantly decreased the nsp1-induced apoptotic rate (Figure 6C–E). Therefore, nsp1-induced autophagy promotes apoptosis, inhibiting cell viability.

### nsp1 induces autophagy in a transgenic mouse model

**Figure 7.**
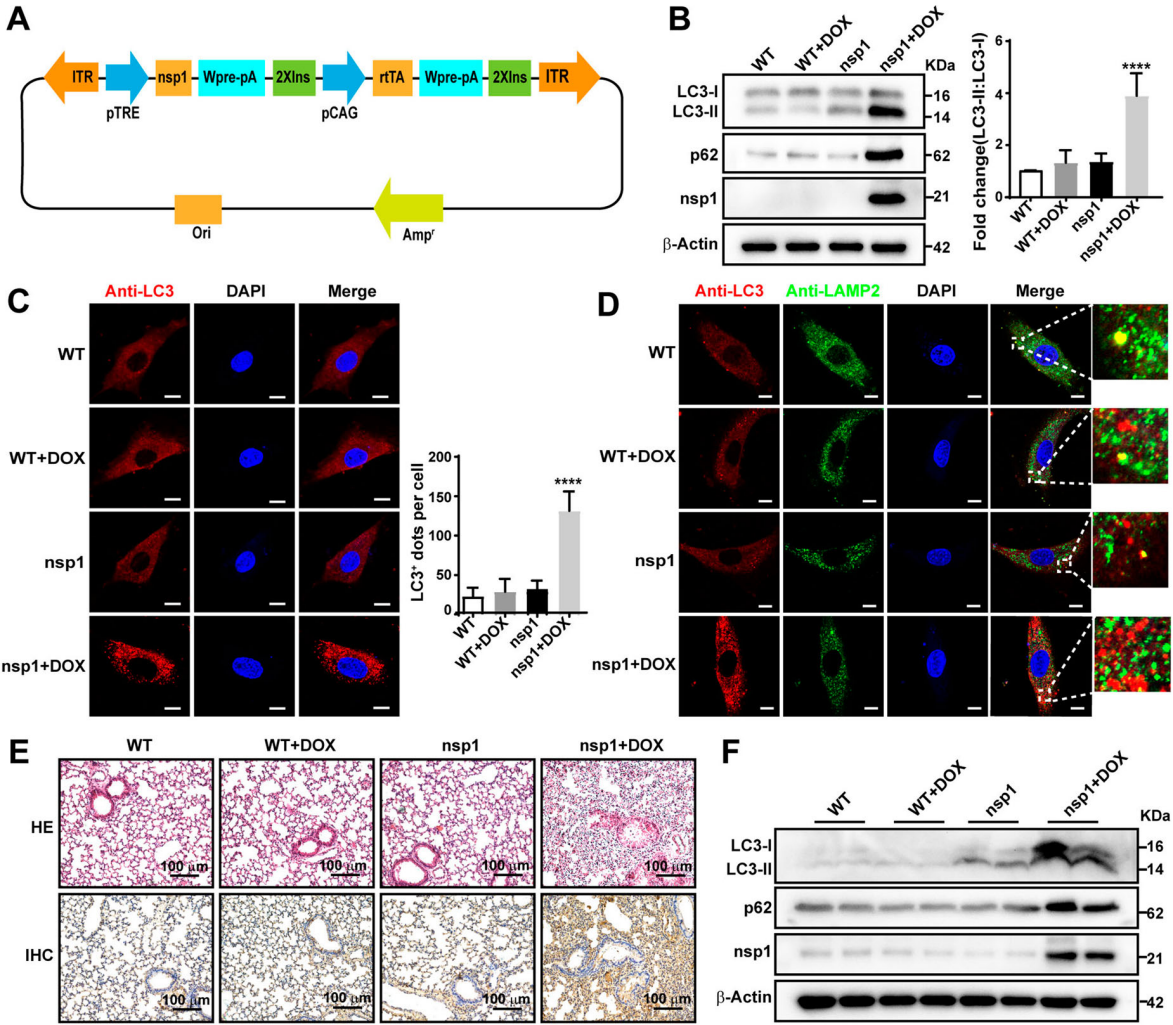
nsp1 induces autophagy in a transgenic mouse model. MEFs were prepared from day 15–18 embryos, and genotype identification was performed. (A) The design strategy of tetracycline-induced nsp1 transgenic mice. (B) MEFs were induced with or without DOX for 3 days, and cell lysates were analyzed by immunoblotting. (C) MEFs were pretreated with or without DOX for 3 days, and cells were fixed and immunostained for LC3. (D) MEFs were fixed and immunostained for LC3 and LAMP2 after DOX induction for 3 days. The co-localization of LAMP2 and LC3 was analyzed by confocal microscopy. (E) Representative images of HE staining analysis and immunohistochemistry staining of p62 in lung tissue. (F) Immunoblotting showed LC3 and p62 levels in the lung tissue. Quantitative analysis of LC3-II:LC3-I ratios was performed with Image J software. The average value in WT was normalized to 1 in the immunoblotting quantitative calculations. LC3^+^ dots in each cell were quantified by Image J software, and at least twenty cells were included for each group. The scale bars are all 10 μm. Data are presented as the mean ± SEM from at least three independent experiments (****p < 0.001).

Finally, we verified the influence of nsp1 on autophagy in a nsp1-transgenic murine model (Figure 7A). MEFs were derived from nsp1-transgenic pregnant mice and cultured *in vitro*. As shown in Figure 7B, the ratio of LC3-II:LC3-I and p62 expression significantly increased in nsp1-positive MEFs treated with DOX. Confocal microscopy revealed that the number of LC3-labelled autophagic vacuoles in nsp1-positive MEFs was significantly increased (Figure 7C). Notably, LC3-labelled autophagic vacuoles did not show significant localization to LAMP2-labelled lysosomes (Figure 7D), indicating that nsp1 inhibited autophagic flux. In addition, the co-localization of Lyso-Tracker-labelled acidic lysosomes and LAMP1-labelled lysosomes was significantly reduced in nsp1-positive MEFs (Figure S7A), indicating that nsp1 affected the acidification of lysosomes in MEFs.

Next, we isolated mouse lung, kidney, and liver tissues and evaluated tissue injury using HE staining. Compared with WT, the alveolar structure was destroyed, bronchial walls were thickened, and lymphocyte infiltration was observed in nsp1-transgenic mice after DOX treatment (Figure 7E; upper panel). Moreover, the kidneys showed significant damage (Figure S7B; upper panel), while the liver did not show a significant change (Figure S7C). Meanwhile, p62 and LC3-II:LC3-I expression in the lung (Figure 7E, down; Figure 7F) and kidney (Figure S7B, down; Figure S7D) of nsp1-transgenic mice was significantly increased by DOX treatment compared to the WT. These data demonstrate that nsp1 can induce autophagy *in vivo*, which is associated with significant tissue injury.

## Discussion

Autophagy is an intracellular catabolic process that plays a pivot in maintaining cellular homeostasis by degrading long-lived proteins and damaged organelles. Studies have shown that viral infection is closely related to autophagy, and notably, autophagy exerts a dual role in fighting viral infections. As a defense system, autophagy delivers viruses or viral proteins for lysosomal degradation. It traffics viral nucleic acids and antigens to lysosomes, activating innate and adaptive immune responses and modulating virus-induced cell death [37]. However, viruses have evolved various strategies to evade or inhibit autophagy and even hijack autophagy to thrive intracellularly [38]. The double-membrane vesicles formed during autophagy can provide a place for virus replication and shelter viral RNA from detection and degradation by the innate immune system [39]. Defective autophagosome maturation causes the accumulation of toxic protein aggregates and damaged organelles, eventually disrupting cellular homeostasis. Additionally, non-functional autolysosomes and hybrid vesicles result from promiscuous fusion with other compartments, contributing to disease pathogenesis [40].

With the outbreak of SARS-CoV-2, the role of autophagy in coronavirus infection has attracted extensive attention worldwide. Coronavirus infection-induced mitochondrial dysfunction is one of important causes of autophagy induction [41]. *Yuan et al.* reported that SARS-CoV-2 nsp1 downregulates mitochondria-related genes, inhibiting mitochondrial function [42]. Our previous study also showed that MERS-CoV nsp1 downregulated the mRNAs of OXPHOS protein genes, leading to mitochondrial dysfunction [10]. Here, we found MERS-CoV nsp1 increased ROS release and reduced ATP production, which can induce autophagy by promoting AMPK phosphorylation and inhibiting mTOR phosphorylation.

Autophagy is a dynamic process in which the fusion of autophagosomes with endosomes or lysosomes is coordinated by SNARE complexes, tethering factors, dynein, and cytoskeletal proteins and promotes the degradation of autophagic cargo [40]. However, many viruses, including coronaviruses, have evolved multiple strategies to inhibit the degradation of autophagic contents, promoting viral replication. SARS-CoV-2 ORF3a inhibits the fusion of autophagosomes with endosomes or lysosomes by interacting with the HOPS complex and suppressing the formation of the STX17-SNAP29-VAMP8 complex [18]. MHV and SARS-CoV-2 impair lysosomal acidity and use lysosomes for egress [43], during which ORF3a and ORF7a play important roles [23]. Beclin1 interacts with ATG14 to promote autophagosome-lysosome fusion. *Gassen et al.* found that MERS-CoV inhibits autophagic flux by promoting ubiquitination and degradation of Beclin1 [28]. Similar to SARS-CoV, MERS-CoV nsp3 interacted with Beclin1 to induce incomplete autophagy by inhibiting the fusion of autophagosomes and lysosomes [20]. Interestingly, the present study demonstrated that nsp1 is also an important protein of MERS-CoV, where it disturbed autophagic flux.

Emerging evidence has revealed that numerous membrane-less structures, including signalling clusters, P-bodies, nuclear speckles, and stress granules, regulate cellular activities, differing from membrane organelles, such as the endoplasmic reticulum and mitochondria [44]. Multivalent interactions between RNAs and RNA-binding proteins promote LLPS, which is the basis for the formation of membrane-less structures, also called “biomolecular condensates” [45]. Phase separation of biological macromolecules exerts a wide range of biological functions, including autophagy regulation and RNA localization [46]. Studies have shown that SARS-CoV-2 N protein promotes viral assembly through phase separation [47]. We recently found that MERS-CoV nsp1 is contained in cellular granules, consistent with the localization of SARS-CoV-2 nsp1 [48,49]. As an endonuclease, nsp1 cleaves mRNAs in granules, including the mRNAs of OXPHOS protein genes and lysosome-related genes, which induce autophagy but impair lysosomal biogenesis and acidification, resulting in the inhibition of autophagic flux.

As a potential causative factor, nsp1 plays an important role in degrading mRNA, inhibiting protein translation, and inhibiting the interferon response in cells. Although nsp1, especially the enzymatic activity, is conserved in coronavirus [10], its specific mechanism varies from virus to virus [30,42]. In α-CoVs, nsp1 of the transmissible gastroenteritis virus inhibits host protein synthesis, which is not associated with the 40S subunit or host mRNA degradation [50]. However, nsp1 of SARS-CoV and SARS-CoV-2 can target the 40S ribosomal subunit to disturb translation at multiple steps of translation initiation [51]. In addition, nsp1 of SARS-CoV, MERS-CoV, and SARS-CoV-2 results in template-dependent cleavage and degradation of mRNAs in infected cells, thereby playing an important role in inhibiting protein synthesis [30]. Therefore, whether nsp1 of other coronaviruses such as SARS-CoV and SARS-CoV-2 also regulates autophagy and the related mechanisms need to be clarified.

Because of the increase in the number of COVID-19 infections, we still face challenges in the prevention and treatment of coronavirus. Currently, the FDA-approved and EUA-authorized anti-SARS-CoV-2 drugs mainly include Remdesivir, immunomodulators (such as Baricitinib, Tocilizumab), and monoclonal antibodies. In addition to blocking the autophagic flux, CQ and its derivative HCQ can inhibit glycosylation of host receptors, spike protein processing and inflammatory responses [52]. In the early stages of the SARS-CoV-2 outbreak, the FDA issued an emergency authorization for the use of CQ and HCQ as experimental treatments for SARS-CoV-2, which was revoked in July 2020 due to serious side effects and a lack of benefits [53]. However, autophagy still attracts extensive attention in the development of anti-coronavirus infection drugs. *Chen F et al.* found that mTOR inhibitors exhibit obvious anti-SARS-CoV-2 activity by using a quantitative systems pharmacological approach [54]. Fluoxetine efficiently inhibited the entry and propagation of SARS-CoV-2 by affecting endolysosomal acidification and cholesterol accumulation in the endosomes [55]. Furthermore, whether more autophagy-related molecules such as AMPK, ULK1, PI3K, Ca2^+^ channels and lysosomes can be the targets for the development of anti-coronavirus drugs needs to be further studied. In addition, Paxlovid is the first anti-COVID-19 drug targeting the 3CL protease [56], indicating nsps of coronavirus can also be used as drug targets. SARS-CoV-2 N protein undergoes LLPS with viral genome RNA, which promotes NFκB activation, inflammatory response [57] and inhibits stress granules, facilitating viral replication and assembly [58], suggesting LLPS may serve as a candidate for the treatment of coronavirus infection. We showed that MERS-CoV nsp1 forms “nsp1-located granules” through LLPS and plays an important role in disturbing mitochondrial function [10] and regulating autophagy. Whether nsp1 can be used as a drug target for the treatment of coronavirus infection remains to be studied.

**Figure 8.**
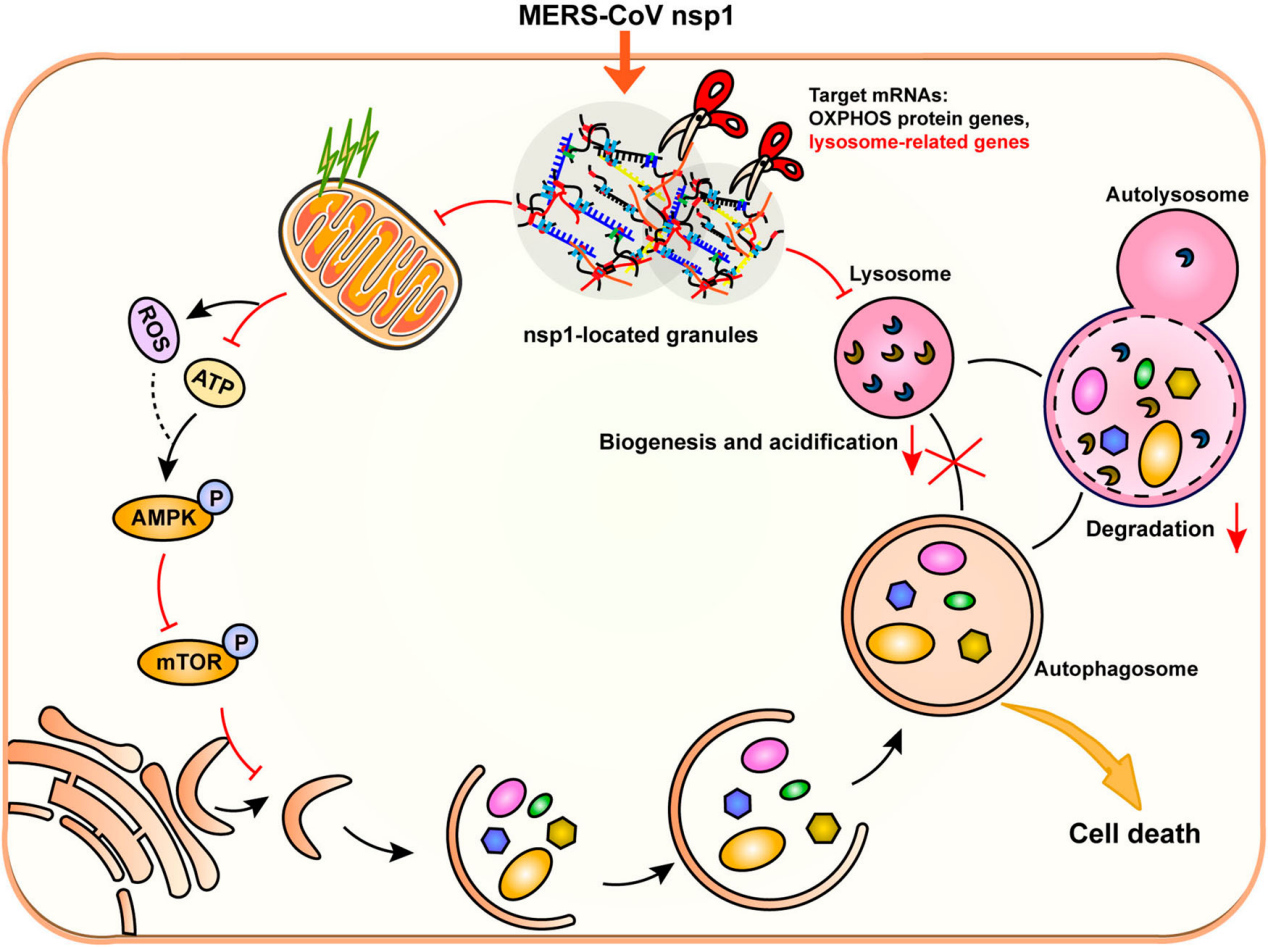
Schematic diagram of the proposed mechanisms involved in nsp1-induced autophagy. nsp1-located granules were rich in RNA-binding proteins and the mRNAs of OXPHOS protein genes and lysosome-related genes. As an endonuclease, nsp1 cleaved the mRNA of OXPHOS protein genes, which disturbed mitochondrial function. The increased ROS and decreased ATP led to the activation of AMPK and the inhibition of mTOR activity, thereby triggering autophagy. On the other hand, nsp1 directly bound to and downregulated the mRNA of lysosome-related genes, blocking the autophagic flux by impairing autophagosome-lysosome fusion and the degradation of autolysosomes. In turn, nsp1-induced autophagy led to cell death.

In summary, MERS-CoV nsp1 was located in granules and interacted with RNA-binding proteins. As an endonuclease, nsp1 cleaved the mRNAs of OXPHOS protein genes and lysosome-related genes, which induced autophagy but affected lysosomal biogenesis and function, thereby blocking autophagic flux. In addition, nsp1-induced autophagy suppressed cell viability, causing cell death (Figure 8). This study clarifies the specific mechanism of MERS-CoV nsp1-induced autophagy, providing new ideas for coronavirus drug development and clinical therapy.

## Supplementary Material

Supplemental Material

## Data Availability

The authors confirm that the data supporting the findings of this study are available within the article and its supplementary materials
