# Supplementary material for: MERS-CoV nsp1 regulates autophagic flux via mTOR signalling and dysfunctional lysosomes
Source: Emerg Microbes Infect. 2022 Oct 26;11(1):2529–43. doi: 10.1080/22221751.2022.2128434 (PMC9621213; doi:10.1080/22221751.2022.2128434)
Supplement: Supplemental Material [file TEMI_A_2128434_SM5469.zip › Revised supplementary material with untrack changes.docx]

**Supplementary material**

**MERS-CoV nsp1 regulates autophagic flux via mTOR signaling and dysfunctional lysosomes**

Yujie Feng, Zhaoyi Pan, Zhihui Wang, Zhengyang Lei, Songge Yang, Huajun Zhao, Xueyao Wang, Yating Yu, Qiuju Han & Jian Zhang*

*Institute of Immunopharmaceutical Sciences,* *School of Pharmaceutical Sciences, Shandong University, Jinan, China*

*Correspondence to Dr. Jian Zhang

Email**:** [zhangj65@sdu.edu.cn](mailto:zhangj65@sdu.edu.cn)

**Supplementary information include:**

Materials and Methods

Figure S1 to S7

Table S1

**Materials and** **Methods**

***Antibodies and reagents***

Mouse monoclonal anti-MERS-CoV nsp1 antibody was prepared in cooperation with KWINBON (Beijing, China). The other antibodies used for this study were: p62 (Proteintech, 18420-1-AP), Beclin1 (MBL, PD017), GFP (ABclonal, AE012), Akt (ABclonal, A17909), p-Akt (ABclonal, AP0140), Mysion VI (Proteintech, 26778-1-AP), Tom1 (Proteintech, 17506-1-AP), GAPDH (Proteintech, 60004-1-Ig), β-Actin (ABclonal, AC026), P70S6K (Proteintech, 14485-1-AP), ATG5 (MBL, M153-3), LAMP2 (Proteintech, 66301-1-Ig), ATP6V1B2 (Proteintech, 15097-1-AP) and CTSD (Proteintech, 21327-1-AP). The following antibodies were purchased from Cell Signaling Technology: LAMP1 (9091), p-AMPK (Thr172; 2535), AMPK (2532), Mouse IgG (5873), p-P70S6K (Thr389; 9234), Lamin A/C (4777T), p-mTOR (Ser2448; 5536), mTOR (2983), TFEB (4240), LC3B (3868), LC3A/B (12741), ERK1/2 (4695), p-ERK1/2 (4370), p-4EBP1 (2855) and EGFR (4267). Fluorochrome-conjugated secondary antibodies were Cy3 goat anti-mouse IgG (ABclonal, AS008), DyLight 549 goat anti-rabbit IgG (Abbkine, A23320), anti-rabbit IgG Alexa Fluor 488 (Abcam, ab181448), and DyLight 488 goat anti-mouse IgG (Abbkine, A232101). The inhibitors used in this study were rapamycin (Selleck, S1039), hydroxychloroquine sulfate (Selleck, S4430), compound C (Selleck, S7840), rotenone (Selleck, S2348), FCCP (Selleck, S8276), oligomycin (Abcam, ab141829), bafilomycin A_1_ (InvivoGen, tlrl-baf1), necrostatin-1 (Selleck, S8037), Z-VAD (OMe)-FMK (Targetmol, T6013), ferrostatin-1 (Selleck, S7243), and NAC (Sigma-Aldrich, A7250). Other reagents used in this study were a multicolor prestained protein ladder (Epizyme, WJ102), protein A/G (Bimake, B23203), phosphatase inhibitor cocktail (Bimake, B15002), protease inhibitor (Bimake, B14002), RNase inhibitor (Beyotime, R0102), Magic Red Cathepsin Assay Kit (ImmunoChemistry, 155), Lyso-Tracker (Beyotime, C1046), and EGF (Life Technologies, PMG8043).

***Plasmids, shRNA, and transfection***

The initial MERS-CoV templates were gifted by Dr. Zhengli Shi (Wuhan Institute of Virology, Chinese Academy of Sciences). We cloned the nsp1 into pCDH and pcDNA3.1. The EGFP-LC3 (11546), mCherry-hLC3 (40827), pEGFP-N1-TFEB (38119), and pBABE-puro mCherry-EGFP-LC3 (22418) vectors were purchased from Addgene. All mutants were generated using PCR-based site-directed mutagenesis as the instructions of the Q5® Site-Directed Mutagenesis Kit (BioLabs, E0554S).

The human BECN1 and ATG5 shRNA lentivirus vectors were generated by ligating the lentivirus vector pLKO.1-puro (Addgene, 19761) with oligonucleotides using T4 DNA ligase (Invitrogen, 15224025), and the correct insertion was confirmed by sequencing. The following sequences were targeted for human BECN1: sh1, CCCGTGGAATGGAATGAGATT; sh2, CTCAAGTTCATGCTGACGAAT; sh3, GCCAGGATGATGTCCACAGAA. The following sequences were targeted for human ATG5: sh1, CCTGAACAGAATCATCCTTAA; sh2, CCTTTCATTCAGAAGCTGTTT; sh3, AGATTGAAGGATCAACTATTT.

All plasmids were transfected into cells using Lipo8000™ (Beyotime, C0533), according to the manufacturer’s instructions.

***Co-immunoprecipitation (Co-IP)***

Cells were collected and washed with pre-cooled PBS. Cell pellets were lysed with NP-40 lysis buffer (25 mmol/L Tris-HCl pH 7.5, 150 mM KCl, 2 mM EDTA, 0.5% NP-40, 1 mM NaF, 1 mM DTT, protease inhibitor and phosphatase inhibitor) for 5 min. After centrifugation (12,000 × g) for 10 min at 4 ℃, supernatants were collected and immunoprecipitated with Protein A/G beads overnight at 4 ℃. The proteins were eluted and analyzed by immunoblotting. For the mass spectrometry assay, protein strips were cut after SDS-PAGE gel separation and Coomassie brilliant blue staining.

***Real-time quantitative PCR(******RT-qPCR)***

Total RNA was extracted using TRIzon Reagent (CWBIO, CW0580), and cDNA was synthesized using a HiFiScript cDNA Synthesis Kit (CWBIO, CW2569). The qPCR Primer Pairs are listed in Table S1. Quantitative PCR was performed according to the instructions of UltraSYBR Mixture (CWBIO, CW2602M) on a Roche LightCycler 480 System. The data were normalized to the 18S rRNA level.

***Reactive oxygen species (ROS) detection***

Intracellular ROS were measured using the fluorescent probe DCFH-DA (Sigma-Aldrich, D6883). Briefly, cells with different treatments were incubated with 10 μM DCFH-DA for 30 min in the dark, washed three times with PBS, and analyzed by flow cytometry.

***ATP detection***

ATP concentration in the cells was detected according to the instructions of the ATP Detection Kit (Beyotime, S0027). Briefly, the lysate was collected and centrifuged at 12,000 × g for 5 min on ice. Before ATP detection, the detection solution was added to a 96-well plate and incubated for 5 min. The lysate was added to the plate, mixed quickly, and read within 30 min. ATP concentration was calculated using an ATP standard curve.

***Cell mitochondrial stress test***

Mitochondrial stress was tested according to the instructions of the Cell Mitochondrial Stress Test Kit. Briefly, the cells were plated on Seahorse culture plates in DMEM supplemented with 10 mM glucose, 1 mM pyruvate, and 2 mM glutamine. The basal oxygen consumption rates were recorded for 30 min. Cells were stimulated with 100 μM oligomycin, 100 μM FCCP, and 50 μM rotenone/antimycin A, and the data were analyzed using Wave software.

***Transmission electron microscope***

Briefly, the cells were fixed with 4% paraformaldehyde and 2.5% glutaraldehyde in 0.1 M sodium phosphate buffer (pH 7.4) for 2 h at 4 °C. Cells were washed and post-fixed in 2% osmium tetroxide for 2 h, dehydrated with a graded series of ethanol, and gradually infiltrated with an epoxy resin. Areas containing cells were thinly sliced and observed using a transmission electron microscope.

***Cell viability assay***

According to the manufacturer’s instructions, cell viability was assessed using the CCK8 assay (Biosharp, BS350A). Briefly, HEK 293T cells were seeded in 96-well plates and cultured for adherence. After different treatments, the cells were incubated with 20 μL CCK8 for 1–2 h at 37 °C. Absorbance was measured at a wavelength of 450 nm.

***Apoptosis detection***

According to the manufacturer’s instructions, apoptosis was determined using the Annexin V/7-AAD apoptosis kit (MultiSciences, 70-AP101-100). Briefly, the attached cells were collected and resuspended in a binding buffer. Then, 100 μL of the cell suspension was stained with 2.5 μL Annexin V for 10 min and then 5 μL 7-AAD for 5 min in the dark. Samples were then analyzed by flow cytometry within 1 h to determine the percentage of apoptotic cells (Annexin V-positive).

***Subcellular fractionation***

Subcellular fractionation was performed as previously described. In brief, cells were collected with lysis buffer (10 mM HEPES, pH 7.9, 10 mM KCl, 0.1 mM EDTA, 0.1 mM EGTA, 1 mM DTT, 0.15% NP-40) on ice and homogenized for 20 strokes. The homogenates were centrifuged at 500 × g for 5 min. The supernatant was collected as the cytoplasmic fraction. The pellet was collected as the nuclear fraction by resuspension in 200 μL of high-salt buffer (20 mM HEPES, pH 8.0, 400 mM NaCl, 1 mM EDTA, 1 mM EGTA, 1 mM DTT, 0.5% NP-40). GAPDH and Lamin A/C were used as control proteins for the cytoplasmic and nuclear fractions, respectively.

**Supplementary Figures**


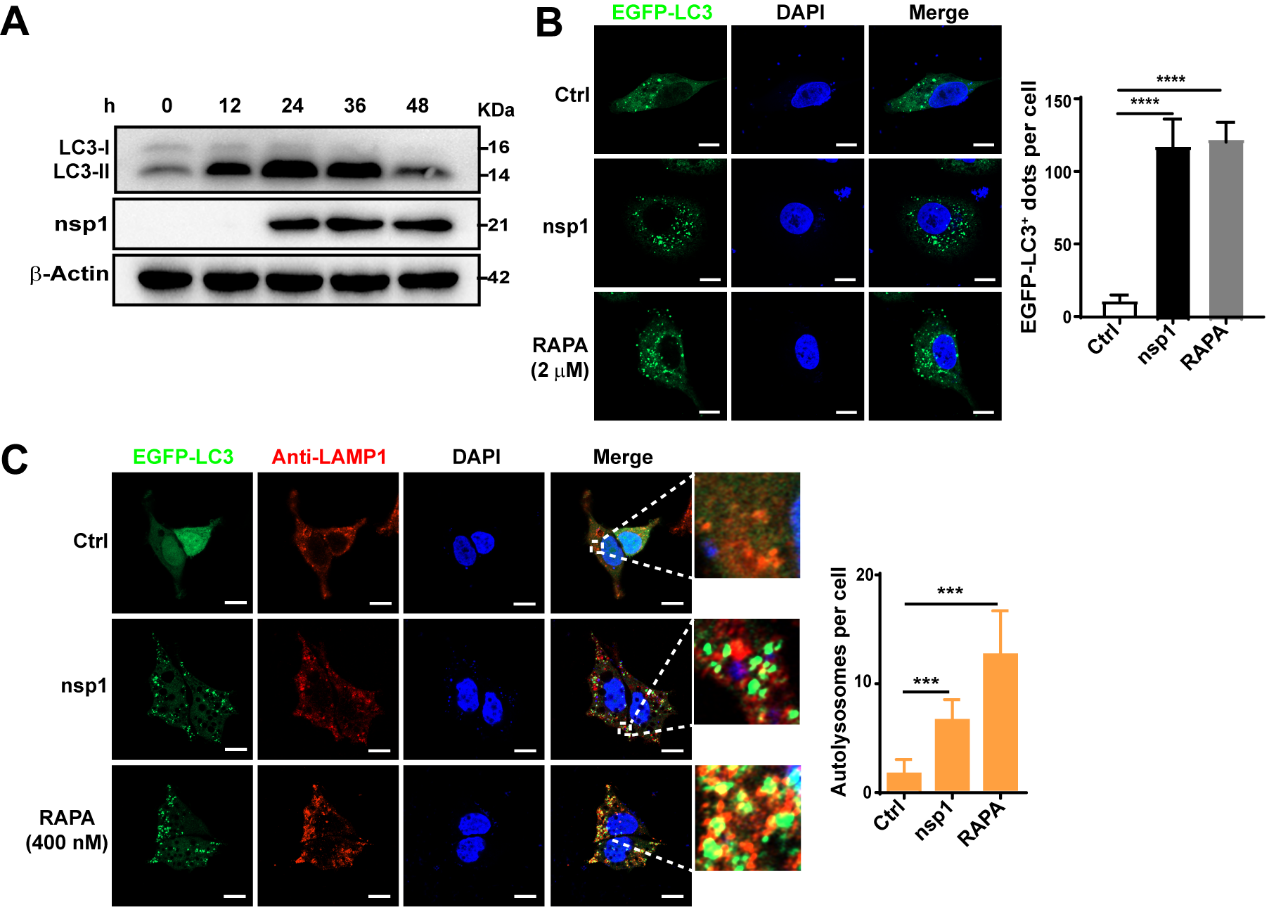


**Figure S1. nsp1 induces autophagy but inhibits autophagic flux.** (**A**) A549 cells were transfected with the nsp1 plasmid for the indicated times, and cell lysates were analyzed by immunoblotting. (**B**) A549 cells were transfected with the indicated plasmids for 24 h, after which confocal microscopy was performed to analyze the EGFP-LC3^+^ dots. (**C**) HEK 293T cells were co-transfected with EGFP-LC3 and the indicated plasmids for 36 h; For the induction of autophagy, HEK 293T cells were transfected with EGFP-LC3 for 30 h and then treated with RAPA for 6 h; the co-localization of LAMP1 and EGFP-LC3 was analyzed by confocal microscopy. Scale bar: 10 μm. The number of EGFP-LC3^+^ dots and autolysosomes in each cell was counted, and at least ten cells were included in each group. Data are presented as the mean ± SEM from at least three independent experiments (***p < 0.001 and ****p < 0.0001).


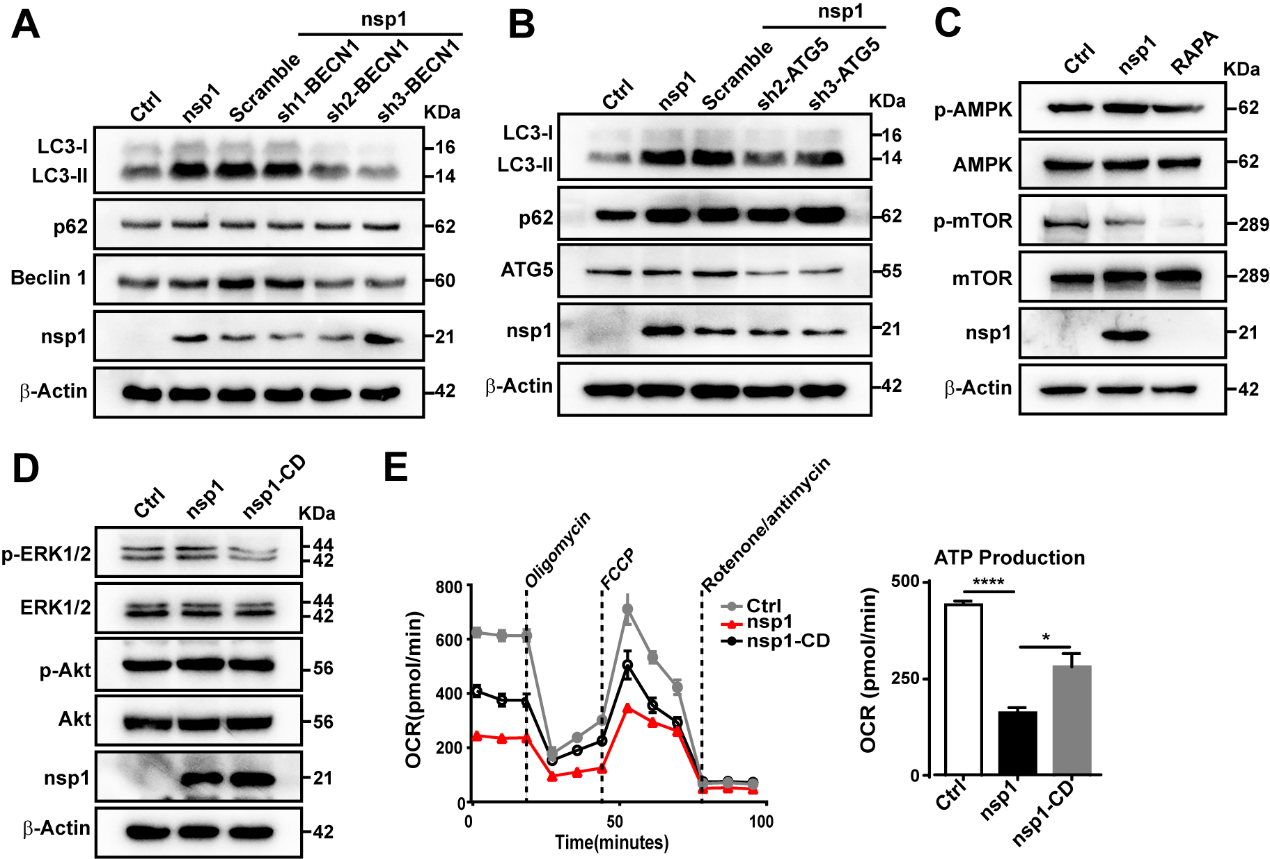


**Figure S2. nsp1 induces autophagy through the AMPK-mTOR pathway.** (**A** and **B**) The indicated plasmids were transfected into Scramble- or (**A**) BECN1 / (**B**) ATG5-knockdown HEK 293T cells for 36 h; cell lysates were analyzed by immunoblotting. (**C**) HEK 293T cells were transfected with the indicated plasmids for 36 h, while Ctrl cells were treated with RAPA for 6 h, and the cell lysates were analyzed by immunoblotting. (**D**) HEK 293T cells were transfected with the indicated plasmids for 36 h, and cell lysates were analyzed by immunoblotting. (**E**) HEK 293T cells were transfected with the indicated plasmids for 36 h, and the oxygen consumption rate of the cells was measured using a Seahorse analyser. Data are presented as the mean ± SEM from at least three independent experiments (*p < 0.05 and ****p < 0.0001).


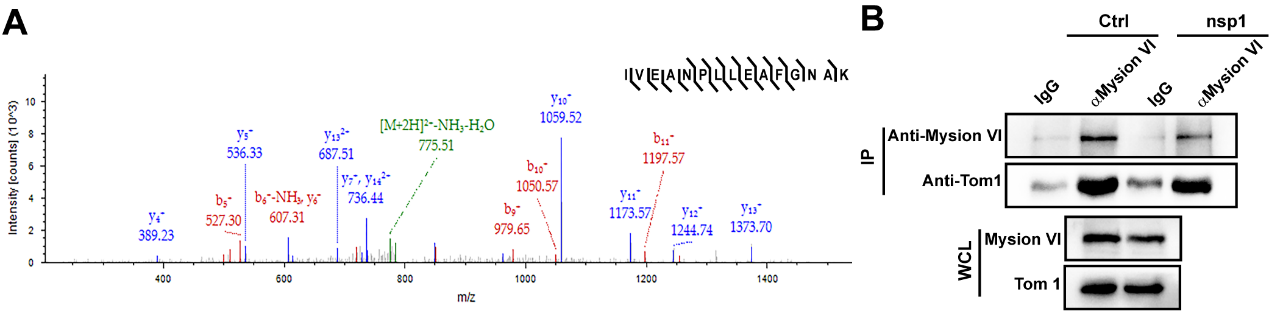


**Figure S3. nsp1 inhibits autophagic flux independent of Myosin VI.** (**A**) The peptide spectrum of Myosin VI was identified by mass spectrometer analysis. (**B**) HEK 293T cells were transfected with the indicated plasmids for 36 h; the interaction of Myosin VI and Tom1 was determined by endogenous Co-IP. Data are presented from at least three independent experiments.


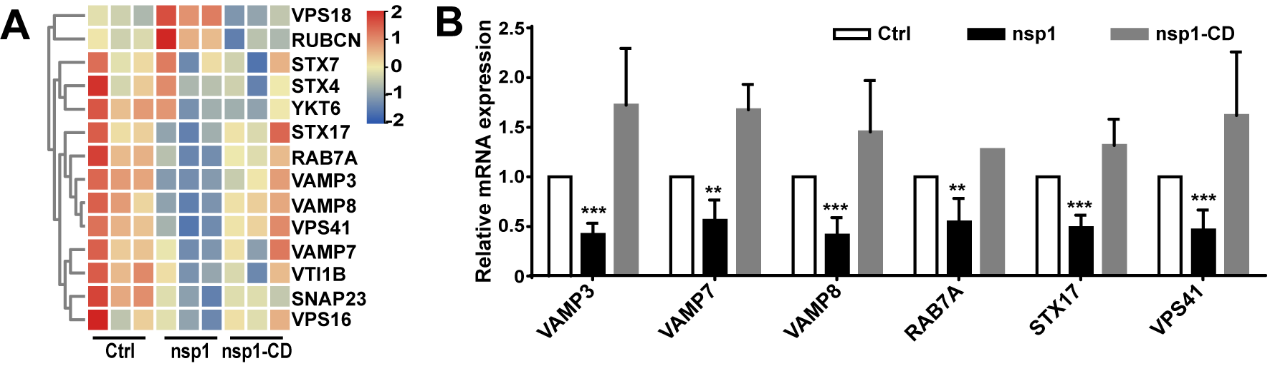


**Figure S4. nsp1 downregulates the expression of lysosome-related genes.** (**A**) Heat map of lysosome-related genes downregulated by nsp1. (**B**) HEK 293T cells were transfected with the indicated plasmids for 24 h, and the transcription levels of the indicated genes were analyzed by RT-qPCR. Data are presented as the mean ± SEM from at least three independent experiments (**p < 0.01 and ***p < 0.001).


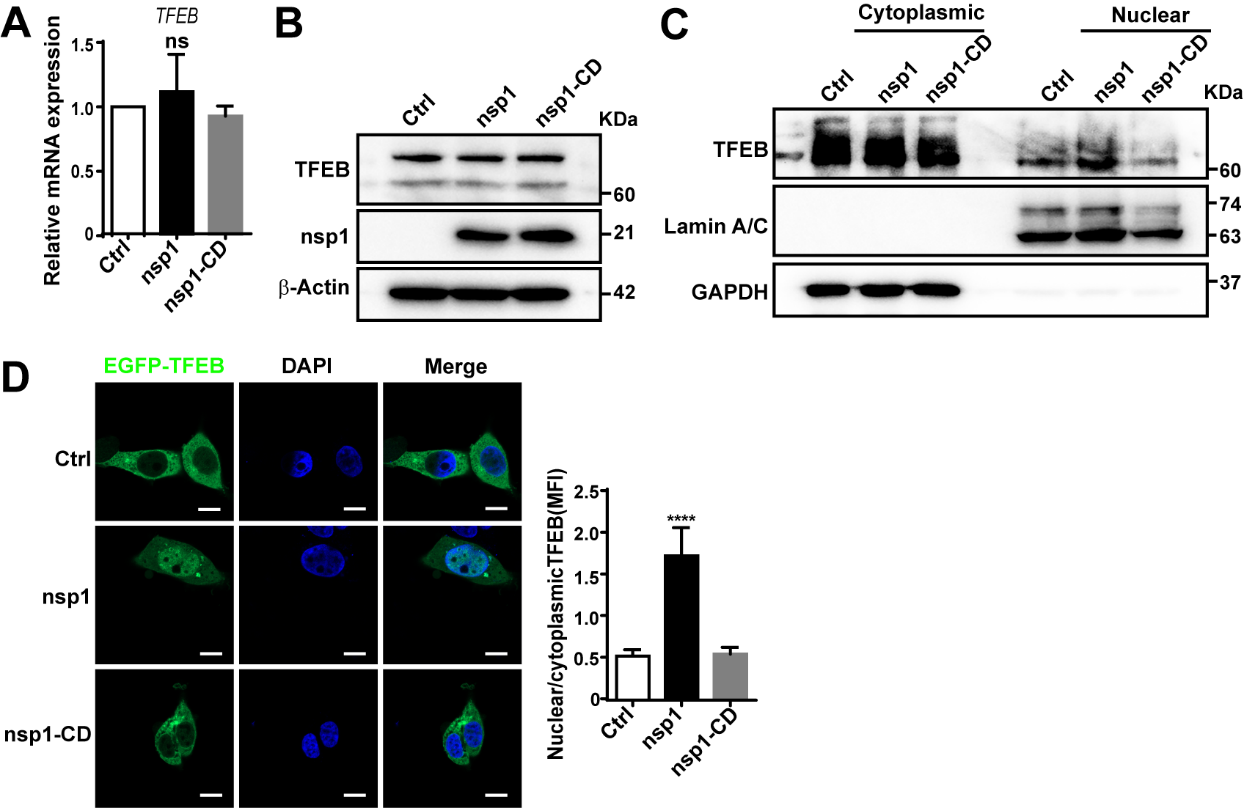


**Figure S5. nsp1 influences lysosomal** **biogenesis and acidification independent of TFEB.** (**A**) HEK 293T cells were transfected with the indicated plasmids for 24 h, and the *TFEB* transcription levels were analyzed with RT-qPCR. (**B**) HEK 293T cells were transfected with the indicated plasmids for 36 h, and cell lysates were analyzed by immunoblotting. (**C**) HEK 293T cells were transfected with the indicated plasmids for 36 h, then the total lysates and nuclear fractions were subjected to immunoblotting. (**D**) HEK 293T cells were co-transfected with the indicated plasmids for 36 h, after which the cytoplasmic and nuclear localization of TFEB were analyzed by confocal microscopy. Scale bar: 10 μm. Data are presented as the mean ± SEM from at least three independent experiments (****p < 0.0001).


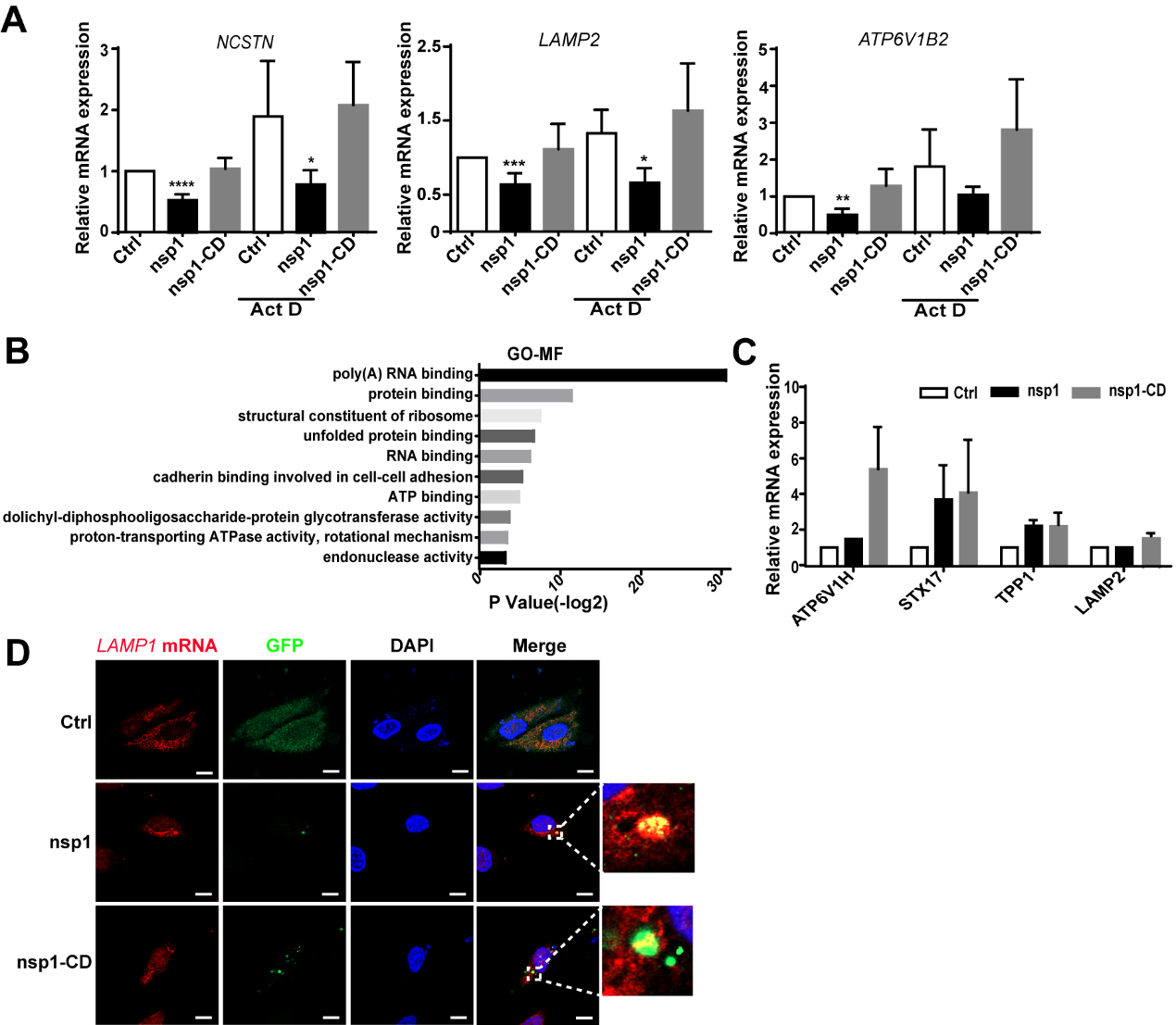


**Figure S6. nsp1 influences lysosomal** **biogenesis and acidification through its enzymatic activity.** (**A**) HEK 293T cells were transfected with the indicated plasmids for 12 h and then treated with Act D (2 μg/mL) or not for 24 h; the transcription levels of the indicated genes were analyzed with RT-qPCR. (**B**) Gene ontology terms of nsp1-located granules enriched with RNA-binding proteins. (**C**) HEK293T cells were transfected with the indicated plasmids for 36 h; the mRNAs that nsp1 directly bound to were detected by RIP-qPCR analysis. (**D**) A549 cells were transfected with the indicated plasmids for 36 h, and the co-localization of *LAMP1* mRNA and nsp1 was analyzed by RNA-FISH. Scale bar: 10 μm. Data are presented as the mean ± SEM from at least three independent experiments (*p < 0.05, **p < 0.01, ***p < 0.001, and ****p < 0.0001).


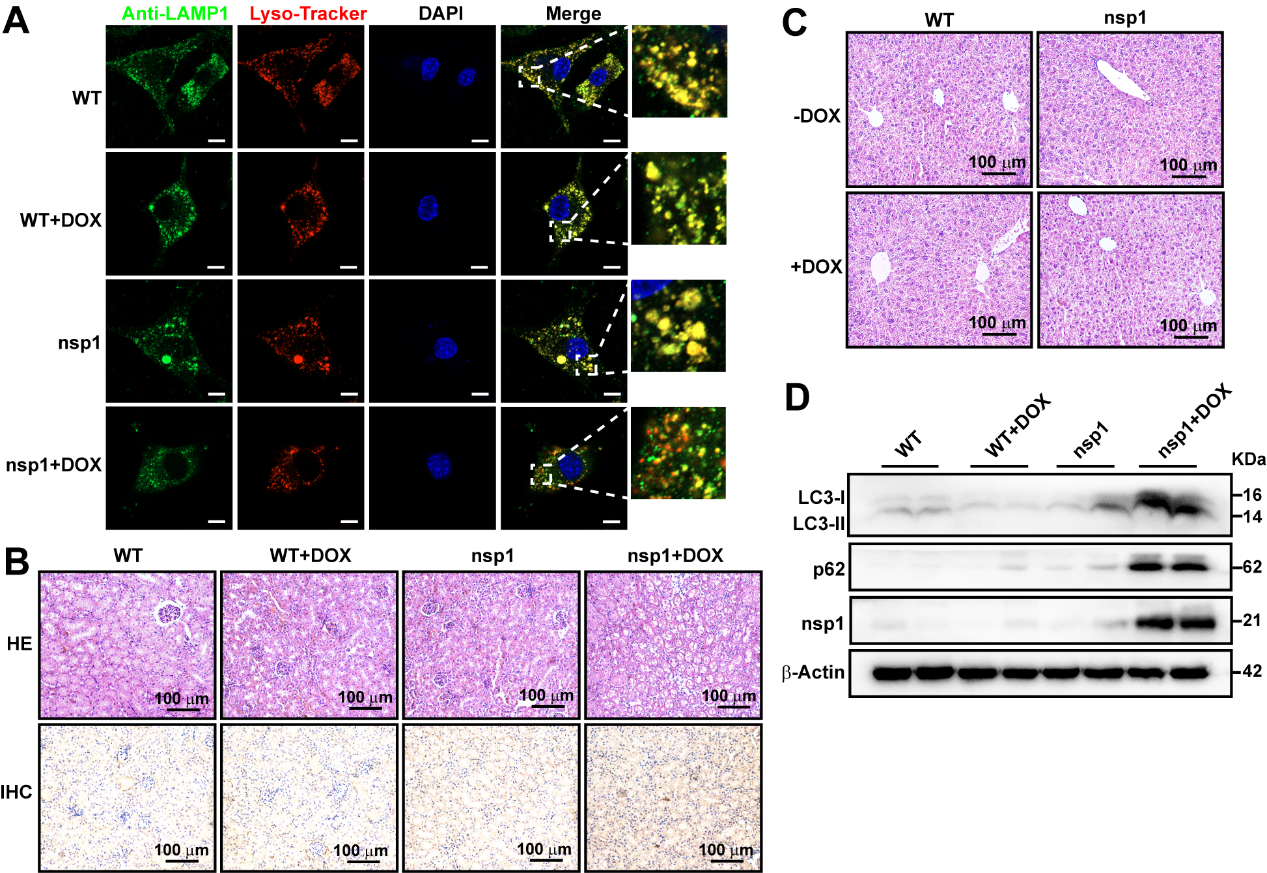


**Figure S7. nsp1 induces autophagy in a transgenic mouse model.** (**A**) MEFs were incubated with Lyso-Tracker for 40 min and then fixed and immunostained for LAMP1 after inducing with or without DOX for 3 days; the co-localization of LAMP1 and Lyso-Tracker was analyzed by confocal microscopy. Scale bar: 10 μm. (**B**) Representative images of HE staining analysis and immunohistochemistry staining of p62 in kidney tissues. (**C**) Representative images of HE staining analysis in liver tissues. (**D**) Immunoblotting showed LC3 and p62 levels in kidney tissues. Representatives from at least three independent experiments.

**Table S1.** **qPCR Primer Pairs list**

| Genes | Forward primers (5'-3') | Reverse primers (5'-3') |
| --- | --- | --- |
| 18S rRNA | CCGGTACAGTGAAACTGCGAATG | GTTATCCAAGTAGGAGAGGAGCGAG |
| LAMP1 | CGTGTCACGAAGGCGTTTTCAG | CTGTTCTCGTCCAGCAGACACT |
| LAMP2 | GGCAATGATACTTGTCTGCTGGC | GTAGAGCAGTGTGAGAACGGCA |
| NCSTN | GGAGGAACCAACTTCAGCGACA | TGCCTGAGGATAGACTGGAACC |
| ATP6V1H | CGGGTCAATGAGTACCGCTTTG | GATACTGGAGCTGAAAGCCACAC |
| ATP6V1C1 | CCACTTGTACGGTGGCTGAAAG | GGGCTGAAGTAGCATTGCTTGG |
| ATP6V1B2 | CTGCTATTGGAGAAGGGATGACC | CTCCAACGACAGCTTTCATGGC |
| HEXB | GATCCATTGTCTGGCAGGAGGT | GGAAGCCAGATGCTGTGACTCT |
| TPP1 | GGTGGCTTCAGCAATGTGTTCC | GAAGTAACTGGATGGTGGCAGG |
| GBA | TGCTGCTCTCAACATCCTTGCC | TAGGTGCGGATGGAGAAGTCAC |
| CTSD | GCAAACTGCTGGACATCGCTTG | GCCATAGTGGATGTCAAACGAGG |
| VAMP3 | GCTCTCTGAGTTAGACGACCGT | CCAGAACAGTAATCCCGATTGCC |
| VAMP7 | CGGTTCAAGAGCACAGACAGCA | ATCCACTTGGGCTTGAGTCTCC |
| VAMP8 | TATGACCCAGAATGTGGAGCGG | ATTTCCGAGCCACCTTCTGCGA |
| RAB7A | GTGATGGTGGATGACAGGCTAG | AGTCTGCACCTCTGTAGAAGGC |
| STX17 | TCGTGGGAAACCTTAGAAGCGG | GCAGCACTGTTGACATGGTCTG |
| VPS41 | CTACATCAGTGGACTTGCACCTC | CAGAAAGTGGCTGGATGATGTCC |
